# Fetal sex differences in placental LCPUFA ether and plasmalogen phosphatidylethanolamine and phosphatidylcholine contents in pregnancies complicated by obesity

**DOI:** 10.1186/s13293-023-00548-1

**Published:** 2023-09-28

**Authors:** Theresa L. Powell, Charis Uhlson, Lana Madi, Karin Zemski Berry, Stephanie S. Chassen, Thomas Jansson, Veronique Ferchaud-Roucher

**Affiliations:** 1 Department of Obstetrics and Gynecology, Division of Reproductive Sciences, University of Colorado Anschutz Medical Campus, Aurora, CO USA; 2 Department of Pediatrics, Section of Neonatology, University of Colorado Anschutz Medical Campus, Aurora, CO USA; 3 Department of Medicine, Division of Endocrinology, Metabolism, and Diabetes, University of Colorado Anschutz Medical Campus, Aurora, CO USA; 4 Nantes Université, CHU Nantes, INRAE UMR 1280 PhAN, CRNH Ouest, 44000 Nantes, France; 5 Nantes Université, INRAE, UMR 1280 PhAN, CHU Hôtel Dieu, HNB1, 1 place Alexis Ricordeau, 44093 Nantes, France

**Keywords:** Sexual dimorphism, De novo placental phospholipid synthesis, Remodeling phospholipid pathway, Maternal obesity

## Abstract

**Background:**

We have previously reported that maternal obesity reduces placental transport capacity for lysophosphatidylcholine-docosahexaenoic acid (LPC-DHA), a preferred form for transfer of DHA (omega 3) to the fetal brain, but only in male fetuses. Phosphatidylethanolamine (PE) and phosphatidylcholine (PC), have either sn-1 ester, ether or vinyl ether (plasmalogen) linkages to primarily unsaturated and monounsaturated fatty acids and DHA or arachidonic acid (ARA, omega 6) in the sn-2 position. Whether ether and plasmalogen PC and PE metabolism in placenta impacts transfer to the fetus is unexplored. We hypothesized that ether and plasmalogen PC and PE containing DHA and ARA are reduced in maternal–fetal unit in pregnancies complicated by obesity and these differences are dependent on fetal sex.

**Methods:**

In maternal, umbilical cord plasma and placentas from obese women (11 female/5 male infants) and normal weight women (9 female/7 male infants), all PC and PE species containing DHA and ARA were analyzed by LC–MS/MS. Placental protein expression of enzymes involved in phospholipid synthesis, were determined by immunoblotting. All variables were compared between control vs obese groups and separated by fetal sex, in each sample using the Benjamini–Hochberg false discovery rate adjustment to account for multiple testing.

**Results:**

Levels of ester PC containing DHA and ARA were profoundly reduced by 60–92% in male placentas of obese mothers, while levels of ether and plasmalogen PE containing DHA and ARA were decreased by 51–84% in female placentas. PLA2G4C abundance was lower in male placentas and LPCAT4 abundance was lower solely in females in obesity. In umbilical cord, levels of ester, ether and plasmalogen PC and PE with DHA were reduced by 43–61% in male, but not female, fetuses of obese mothers.

**Conclusions:**

We found a fetal sex effect in placental PE and PC ester, ether and plasmalogen PE and PC containing DHA in response to maternal obesity which appears to reflect an ability of female placentas to adapt to maintain optimal fetal DHA transfer in maternal obesity.

**Supplementary Information:**

The online version contains supplementary material available at 10.1186/s13293-023-00548-1.

## Highlights

- Ether and plasmalogen phosphatidylcholine (PC) and phosphatidylethanolamine (PE) are considered DHA reservoirs and whether their metabolism in placenta and DHA transfer to the fetus are altered in obesity is unexplored, especially regarding the fetal sex.
- We found that in obesity, (1) pregnancies with male fetuses decrease ester PC with DHA and ARA in placentas but not plasmalogen PC species and the levels of ester, ether and plasmalogen PC and PE with DHA is decreased in fetal umbilical circulation, and (2) pregnancies with female fetuses decrease ether and plasmalogen PE with DHA and ARA in placentas and maintain the levels of ester, ether and plasmalogen PC and PE with DHA in fetal circulation.
- Both males and females have compensatory mechanisms that attempt to maintain an adequate supply of DHA species, particularly for brain development, in obesity, however, the male placental adaptation failed to maintain fetal levels to those found in normal weight pregnancies.

**Supplementary Information:**

The online version contains supplementary material available at 10.1186/s13293-023-00548-1.

## Plain language summary

Docosahexaenoic acid (DHA) is a critical omega 3 long chain polyunsaturated fatty acid (LCPUFA) for fetal brain development. We have recently reported that maternal obesity reduces placental transport capacity for LysophosPhatidylCholine-DHA (LPC-DHA), a preferred form for transfer of DHA to the fetal brain, but only in male fetuses. Other important lipids, the plasmalogen phosphatidylcholine (PC) and phosphatidylethanolamine (PE) are considered DHA reservoirs, but its roles in the maternal–fetal unit are largely unexplored. We examined these lipid species in maternal and fetal circulation and in placental tissue to uncover potential novel roles for ether and plasmalogen lipids in the regulation of placenta delivery of these vital nutrients in pregnancies complicated by obesity depending of fetal sex. We demonstrated for the first time, that female fetuses of obese mothers decrease placental ether and plasmalogen PE containing DHA and arachidonic acid (ARA, omega 6), and show a high fetal–placental adaptability and placental reserve capacity that can maintain the PC-LCPUFA synthesis and the transfer of these crucial species to the fetus to preserve brain development. Our study also demonstrated that male fetuses, in response to maternal obesity, reduce the placental ester PC species containing DHA and ARA and reduce the ether and plasmalogen PE reservoir of DHA and ARA in fetal circulation. Our findings support a fetal sex effect in placental ester, ether and plasmalogen PE and PC containing DHA in response to maternal obesity which appears to reflect an ability of female placentas to adapt to maintain optimal fetal DHA transfer in maternal obesity.

**Supplementary Information:**

The online version contains supplementary material available at 10.1186/s13293-023-00548-1.

## Introduction

The worldwide prevalence of maternal obesity continues to increase and affects not only maternal health, but also negatively impacts fetal growth and development by modulating placental function [1]. Placental dysfunction has been recently shown to be associated with changes in fat mass at birth and with risks of metabolic disorders later in life [2]. Pre-gravid maternal obesity is associated with an elevated risk of obesity in boys from age one [3]. In addition, pregnancies complicated by overweight and obesity are associated with long-term cognitive issues in the children, such as attention deficit and hyperactivity disorder (ADHD) [4] and autism spectrum disorder (ASD) [5] with male children being more frequently affected than female children [6]. For example, boys aged 7–11 years have reduced hippocampal volume when their mothers were obese during pregnancy but this was not observed in girls [7]. Similar sex differences in brain development have been described in children of mothers with gestational diabetes mellitus (GDM) [8]. Numerous studies found correlations between maternal obesity and child neurodevelopment disorders [9, 10] suggesting in utero programming of brain development. However, the mechanisms linking maternal obesity in pregnancy and impaired neurodevelopment in children remain unclear.

Long chain polyunsaturated fatty acids (LCPUFA), such as docosahexaenoic acid (DHA, C22:6n-3), are considered essential nutrients for fetal development, particularly for the brain. Supplied by the mother’s dietary intake, DHA is preferentially taken up by the placenta [11, 12], metabolized and transferred to the fetus through the trophoblast basal plasma membrane and the fetal endothelium mediated by specific lipid transporters [13, 14]*.* Maternal n-3 LCPUFA deficiency has been reported in pre-gestational obese women [15] and is associated with decreased visual acuity, hypertension, diabetes, ADHD and ASD in the offspring [16]. Whether the DHA deficit in maternal circulation of obese mothers is related to her own metabolism and thus results in decreased transplacental supply of LCPUFAs is not well known. If lower maternal supply was the cause of decreased LCPUFA transfer through the placenta, the fetal sex differences on neurodevelopment are more difficult to explain. However, placental function is dependent on fetal sex [17] and may contribute to differences in delivery of DHA to the developing fetus.

DHA and arachidonic acid (ARA, C20:4n-6) are highly abundant in circulating phospholipids, specifically phosphatidylcholine (PC) and phosphatidylethanolamine (PE), and increase in the maternal circulation over gestation. Whereas PE displays the most significant increase during pregnancy compared to the other phospholipid classes [18], the level of DHA and ARA is higher in PC than in PE. PC is synthesized de novo by two pathways in the endoplasmic reticulum: (1) the Kennedy pathway, a part of the cytidine diphosphocholine (CDP-choline) pathway, which produces the majority of cellular PC from dietary choline and non-esterified fatty acids (NEFAs) and at a lesser extend the conversion of PE to PC through the phosphatidylethanolamine methyltransferase (PEMT) pathway; (2) the acyl-chain composition of PC can be modified through Land's cycle (remodeling pathway), by replacement of fatty acids at the sn-2 position by the combined action of the calcium-independent Group VIA phospholipase A2 (iPLA2) and reacylation by lysophosphatidylcholine acyltransferase (LPCAT) [19].

We have previously demonstrated that levels of non-esterified DHA and lysophosphatidylcholine containing DHA (LPC-DHA) are lower in placentas of obese women carrying a male fetus and reduced LPC-DHA levels were also found in cord blood, indicating reduced delivery to the fetus. This was in spite of an up-regulation of the protein expression of MFSD2a, LPC-DHA transporter, in the basal syncytiotrophoblast plasma membrane of male placentas, suggesting that the adaptation in transporter expression was insufficient to maintain normal DHA transfer to the fetus [20]. We speculate that reduced DHA transfer to the fetal circulation may negatively impact brain development in male fetuses of obese mothers.

The physicochemical characteristics of phospholipids affect the structure and function of cell membranes. PC and PE can have either ester (common diacyl phospholipids), ether (O = alkyl glycerophospholipid) or vinyl ether (P = plasmalogen glycerophospholipid) linkages to primarily unsaturated and monounsaturated fatty acids at the sn-1 position of the glycerol backbone. The phospholipid fraction in most cell membranes contains 15–20% plasmalogens with vinyl ether linked to sn-1 position [21]. Despite the high abundance of plasmalogens in cell membranes in many organs such as brain, heart and kidney [22], the role of ether and plasmalogen linkages in phospholipids remains unclear and literature on lipidomic analysis often do not specifically differentiate plasmalogen phospholipids from ether linked phospholipids. Because plasmalogens are preferentially enriched in ARA and DHA at the sn-2 position [23], they may function as reservoirs for these important LCPUFAs. Indeed, due to the presence of the vinyl ether at the sn-1 position, the plasmalogens cannot be hydrolyzed by phospholipase A1. However, LCPUFAs at the sn-2 position can be released from plasmalogens by PLA_2_ hydrolysis during the remodeling process [24]. Release of ARA is vital as a precursor for important lipid signaling molecules (prostaglandin, leukotriene, thromboxane) and DHA is mainly esterified into ester forms of phospholipids such as PC, which are crucial components of cell membranes in the brain and neural tissues, or transported as LPC-DHA by MFSD2a [25].

Ether phospholipids and particularly plasmalogens are thought to also act as endogenous antioxidants that protect critical cell membrane components such as LCPUFA from oxidative stress [3, 26]. A protective role for LCPUFA containing plasmalogens in atherosclerosis was demonstrated in LDLR−/− mice [27] and APOE-deficient mice [28]. Consistent with these data, lower levels of plasmalogen PC of HDL are associated with coronary disease [29] and decreased serum ether lipid levels have been implicated in hypertension and obesity [30, 31]. In addition, lower plasmalogen proportion in phospholipids is associated with neurodegenerative diseases such as Alzheimer, with specific decreases in plasmalogen PC and PE containing DHA [32]*.* Conversely, serum plasmalogen levels increase in men with aerobic training and in patients after a healthy dietary intervention [33, 34]. Although altered circulating levels of these compounds are associated with higher risks to develop cardiovascular and degenerative diseases, few clinical studies have investigated their role over healthy pregnancy and in pregnancy complicated by obesity and their impact on fetal development. One study has observed increased plasma ether PC containing DHA (PC O-16:0_22:6) in healthy women at 26–28 weeks of pregnancy compared to postnatal 4–5 years after delivery [18]. We have recently identified some ether linked phospholipid species in human placenta across gestation and found PE containing LCPUFA, such as PE O-16:1_22:6 and PE O-16:1_20:4, are enhanced in the third trimester compared to the first and second trimesters [35]. These data suggest a potential implication of those phospholipids in the placental LCPUFA metabolism and transfer to the fetus. A specific role of placental ether and plasmalogen PC and PE containing DHA and ARA particularly in the context of maternal obesity as how they may relate to transfer to the fetus depending of fetal sex is currently unknown. We therefore hypothesized that ester, ether and plasmalogen linked PC and PE containing DHA and ARA are reduced in maternal–fetal unit in pregnancies complicated by obesity and these differences are dependent on fetal sex.

## Materials and methods

### Subjects and sample collections

Pregnant women were enrolled before delivery to donate their blood, placenta and umbilical cord blood to a data/bio-repository for use in multiple research studies under a protocol approved by the Institutional Review Board at University of Colorado, Denver (COMIRB 14-1073). All participants gave their informed written consent for the use of their biological samples and protected health information. Inclusion criteria for the repository included information on pre-pregnancy/early pregnancy BMI, ultrasound confirmation of gestational age at 14–18 weeks, singleton pregnancy, and maternal age 18–45 years. Exclusion criteria included concurrent inflammatory, vascular, or metabolic disease, current use of tobacco, street drugs, or medications, fetal malformations, history of pregnancy loss or pre-term delivery, any pregnancy pathology (gestational diabetes, hypertension, pre-eclampsia) other than elevated BMI. Anonymized biological samples such as maternal blood, placenta and umbilical cord vein and artery were transferred to the lab for preparation and storage at − 80 °C for batch analysis. Pregnant women involved in the current study were either normal weight (BMI, range 18.5–24.9 kg/m^2^), i.e., control group (*n* = 16; *n* = 9 females, *n* = 7 males) or obese (BMI, range 30–45 kg/m^2^) (*n* = 16; *n* = 11 females, *n* = 5 males). Maternal and infant characteristics for study subjects are shown in Additional file 1: Table S1A and have been previously reported [20]. For placental protein expression analysis, placentas were collected immediately after delivery and homogenized in buffer D (250 mM sucrose, 10 mM hepes, pH 7.4), including a 1:100 dilution of both phosphatase and protease inhibitors (Sigma-Aldrich, St. Louis, MO) and stored at − 80 °C until batch processing. The number of studied placentas was increased to 38 (control group *n* = 18; *n* = 10 females and *n* = 8 males, obese group *n* = 20; *n* = 10 females and *n* = 10 males) to increase power to allow studies of the influence of fetal sex (Additional file 1: Table S1B).

### Quantification of protein expression by western blot and simple western

Total protein content of placental homogenates was determined using BCA assay (Pierce BCA Protein Assay Kit, Thermo Scientific, MA, USA). Twenty µg total protein was loaded and separated on Bis–Tris gels (4–20%) using a pre-cast gel systems (Bio-Rad) as previously described [36]. After electrophoresis and transfer onto a PVDF membrane (Bio-Rad Laboratories Inc.), we stained for total protein using Amido Black Stain (Sigma-Aldrich). Incubation of primary antibody LysoPhosphatidylCholine Acyl Transferase 4 (LPCAT4), PhosphoLipase A_2_ Group IVC (PLA_2_G4C or iPLA_2_) and 1-acylglycerol-3-phosphate O-acyltransferase 4 (AGPAT4) was carried out overnight at 4 °C (details on primary antibodies are provided in Additional file 1: Table S2), and secondary antibody (peroxidase labeled anti-Rabbit IgG, diluted 1:3000, Cell Signaling Technology (Danvers, MA)) for 1 h at room temperature as previously depicted [35]. Immunolabeling was made visible with SuperSignal West Pico Plus detection solution (Thermo Scientific) in a G:Box ChemiXL1.4 (SynGene, Cambridge, UK). Densitometry analysis of target protein bands was performed with GeneTools (SynGene) and target protein expression was adjusted for Amido Black staining to account for any variation in protein loading and transfer.

We also used the JESS (capillary-based immunoblotting, Simple Western with total protein normalization—ProteinSimple) [37] to measure the abundance of target proteins: fatty acyl-CoA reductase 1 (FAR1), 1-acylglycerol-3-phosphate O-acyltransferase 2 (AGPAT2), and glycerol-3-phosphate O-acyltransferase 3 (GPAT3) (Additional file 1: Table S2). We ran the plates based on the recommended manufacturer’s settings (separation voltage of 375 for 25 min) with the placental homogenates at a 1 µg/µl protein concentration per well. An equalizer sample was used between each plate to correct for variations, and positive controls were run with each antibody to confirm the presence of the target.

### Analysis of phosphatidylcholine and phosphatidylethanolamine species containing LCPUFA in plasma and placental tissue by LC–MS/MS

Phosphatidylcholine (PC) and phosphatidylethanolamine (PE) were extracted and analyzed in placental homogenates and plasma samples [maternal vein (MV), umbilical vein (UV) and artery (UA)], by LC–MS/MS in the Mass Spectrometry Lipidomics Core facility at the University of Colorado using a protocol previously described [20, 36]. Briefly, 1 µl of a commercial internal standard mixture containing the major phospholipid classes (SPLASH Lipidomix, from Avanti Polar Lipids, Inc.) was added to 50 µl of each plasma and placental homogenate sample and lipids were extracted according to the method of Bligh and Dyer [38]. LC–MS/MS system equipped with a HPLC system (Shimadzu, Kyoto, Japan) with a normal phase chromatography (silica HPLC column, Ascentis, 150 × 2.1 mm, 5 µm, Supelco, Bellefonte, PA) and a 5500 QTRAP mass spectrometer (SCIEX, Framingham, MA) was used. PC and PE containing esterified ARA or DHA chains were analyzed by scanning precursor ions of the *m/z* 303 or of the *m/z* 327 product anion, respectively. Results were analyzed using MultiQuant software (SCIEX) and all detected ARA and DHA containing PC and PE results are reported as the ratio between the integrated area of each analyte and the integrated area of the corresponding internal standard (IS) for PC class (PC 15:0_[^2^H_7_]18:1 and LPC [^2^H_7_]18:1) and PE class (PE 15:0_[^2^H_7_]18:1 and LPE [^2^H_7_]18:1).

In order to distinguish vinyl ether (P = plasmalogen) from 1-O-alkyl (O = ether) linkages at the sn-1 position of PC and PE species, acid labile plasmalogens were hydrolyzed by exposure to 12N HCl fumes [39]. This was achieved by drying down the placenta total lipid extract under a stream of nitrogen followed by exposure to hydrochloric acid fumes for 1 h. Upon acid hydrolysis, the vinyl ether bond of the plasmalogens was hydrolyzed but the 1-O-alkyl or 1-acyl phospholipid species remained intact. The lipids were reconstituted in initial HPLC conditions for LC–MS/MS analysis and plasmalogen identity was determined by disappearance of the molecular species by comparing pre- vs post-HCl exposed samples.

### Statistical analyses

All statistical analyses were performed using GraphPad Prism version 9.01. Results are expressed as mean ± SD for each sample type; maternal vein (MV), placenta, umbilical vein (UV) and artery (UA). Chi-square test was used to compare qualitative data in obese and control groups. Data distribution was assessed by the Shapiro–Wilk normality test. All lipid variables were compared between two independent groups, control vs obese groups and separated by fetal sex, in each sample. We applied the Benjamini–Hochberg false discovery rate adjustment to account for multiple testing. All tests were two-sided. A significance threshold set at q ≤ 0.05 was used for the targeted lipidomic analysis [40]. Correlation analysis with spearman test was performed to assess correlations between maternal obesity and ether and plasmalogen PC and PE species in placentas and in umbilical cord. *p* values < 0.05 were considered as statistically significant.

In addition, for each phospholipid variable showing a statistical difference between groups, effect size was calculated as following: ((Mean PC ob − Mean PC ctr)/ mean PC ctr) × 100).

## Results

Clinical parameters of mothers and their infants are provided in Additional file 1: Table S1A and separated by fetal sex in Additional file 1: Table S1B. By design, maternal BMI in the obese group were greater than in the normal weight group. Maternal age at delivery, gestational age, placenta and birth weights were not significantly different between the obese vs control groups for either sex.

### Plasma from obese mothers have lower levels of sn-1 ether and plasmalogen PC and PE containing DHA but not ARA

We analyzed all the species of sn-1 ester (common form), ether and plasmalogen PC and PE containing DHA and ARA in maternal venous plasma, in placenta and in umbilical cord venous and arterial plasma by LC–MS/MS.

**Fig. 1:**
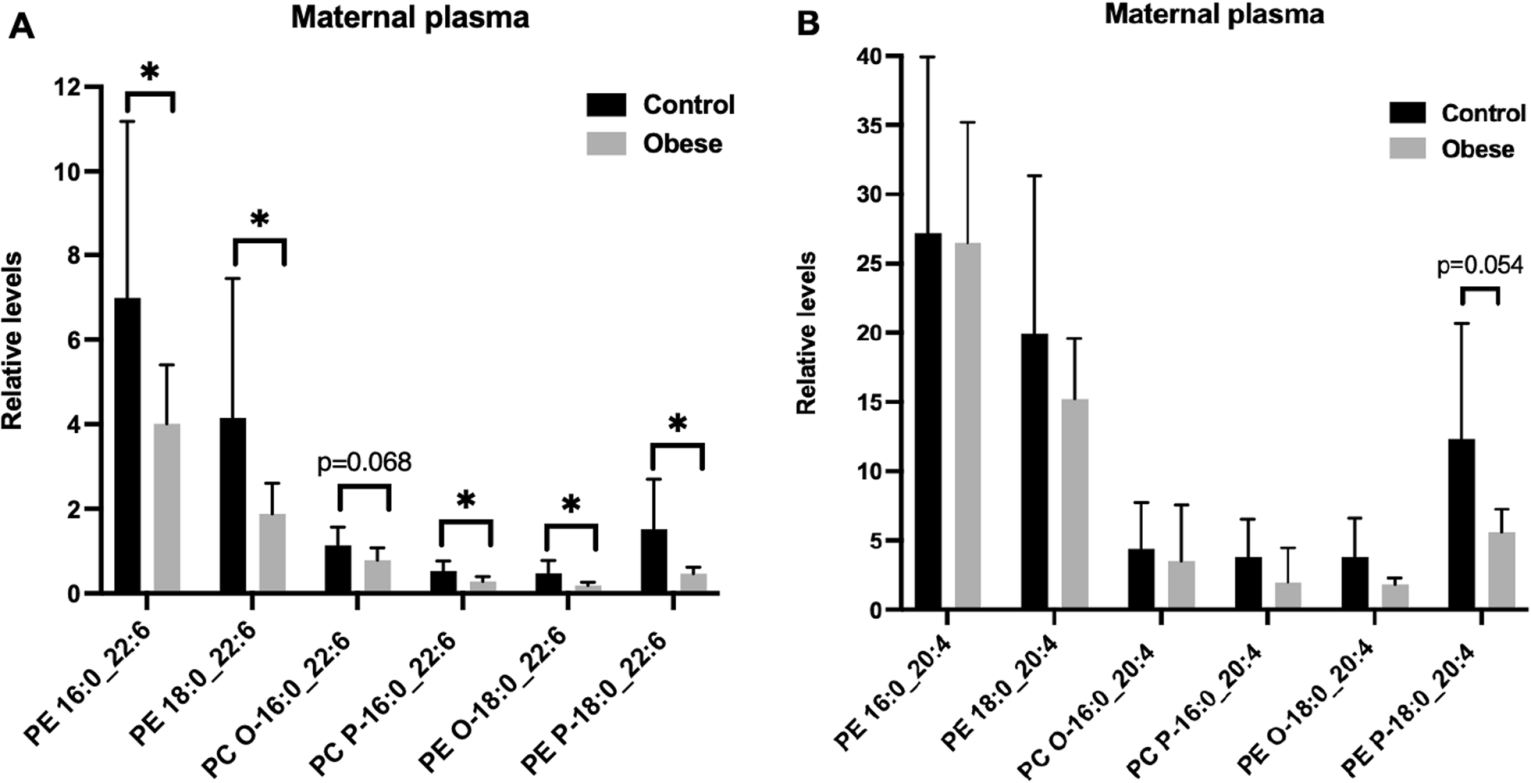
Differences in PC and PE containing DHA and ARA variables in maternal plasma between the two groups (control/obese) of mothers including both male and female fetuses. **A** Significant differences in PC and PE containing DHA in maternal plasma between control and obese groups. **B** PC and PE species containing ARA were not changed between control and obese mothers. Relative levels are expressed in mean ± SD and significant differences pass at false-discovery rate-adjusted p value (Benjamini–Hochberg false discovery rate adjustment to account for multiple testing). *p < 0.05

Metabolic profile of obese mothers was characterized by lower ester, ether and plasmalogen PC and PE species containing DHA compared to normal weight mothers. Differences achieving statistical significance are presented in Fig. 1A. Those PC and PE species with ARA were not affected by maternal obesity (Fig. 1B).

Then, we studied the influence of the fetal sex on maternal circulating phospholipids, by comparing the levels of all PC and PE species containing DHA and ARA detected in plasma of obese women to those in plasma of normal weight women carrying either a male or a female fetus. Interestingly, only LPE 22:6 was significantly decreased in obese mothers compared to normal weight mothers carrying a male (*p* = 0.025, reduced by 50%) (Additional file 1: Table S3).

### Fetal sex differentially influences the levels of PC and PE containing DHA and ARA in placenta of obese mothers

The evaluation of fetal sex differences in the placenta revealed distinct changes in pregnancies complicated by maternal obesity compared to healthy pregnancies. We observed decreases in the ester linked PC containing DHA and ARA in male placentas whereas the levels of almost all PE containing DHA and ARA including those with sn-1 ether and plasmalogen linkages were reduced in female placentas (Table 1). These findings suggest differing mechanisms for handling of LCPUFA in phospholipids in the placenta of male and female fetuses exposed to the obesogenic environment. These sex differences are all the more relevant because the levels of those phospholipid species were similar between the female and male placentas of mothers with normal weight (control) (Additional file 1: Figure S1). However, the specific lipids species impacted was highly influenced by fetal sex with the male placentas primarily and profoundly reduced in ester PC forms by 60–92% while the female placentas demonstrated significant decreases of 51–84% in PE forms.

**Table 1:** Profile of phosphatidylcholine (PC) and phosphatidylethanolamine (PE) containing DHA and ARA in placenta of obese (*n* = 16) compared to normal BMI women (*n* = 16) regarding the fetal sex

	Placenta					
	Female			Male		
Relative level/µg prot	Ctr (*n* = 9)	Ob (*n* = 11)	*p* value (FDR)	Ctr (*n* = 7)	Ob (*n* = 5)	*p* value (FDR)
PC-DHA						
PC 16:0_22:6	0.03 ± 0.02	0.01 ± 0.008	0.126	0.030 ± 0.010	0.012 ± 0.005	**0.045**
PC O-16:0_22:6	0.01 ± 0.01	0.006 ± 0.004	0.179	0.012 ± 0.007	0.004 ± 0.002	0.129
PC P-16:0_22:6	0.009 ± 0.007	0.004 ± 0.003	0.126	0.008 ± 0.005	0.003 ± 0.001	0.202
PC 18:0_22:6	0.02 ± 0.02	0.009 ± 0.005	0.101	0.025 ± 0.015	0.008 ± 0.003	**0.039**
PC 18:1_22:6	0.01 ± 0.007	0.004 ± 0.004	0.098	0.010 ± 0.006	0.005 ± 0.002	0.157
PC O-18:0_22:6	0.01 ± 0.01	0.003 ± 0.002	0.126	0.010 ± 0.008	0.003 ± 0.001	0.129
PC O-18:1_22:6	0.02 ± 0.02	0.004 ± 0.002	0.101	0.013 ± 0.010	0.003 ± 0.002	0.101
PC 20:4_22:6	0.005 ± 0.004	0.003 ± 0.002	0.284	0.003 ± 0.002	0.002 ± 0.002	0.157
LPC 22:6	0.0007 ± 0.0003	0.0004 ± 0.00008	0.098	0.001 ± 0.0001	0.0002 ± 0.00005	**0.039**
PC-ARA						
PC 16:0_20:4	0.76 ± 0.58	0.24 ± 0.18	0.098	0.74 ± 0.49	0.06 ± 0.01	**0.029**
PC 16:1_20:4	0.02 ± 0.01	0.001 ± 0.001	0.101	0.02 ± 0.02	nd	
PC O-16:0_20:4	0.14 ± 0.11	0.05 ± 0.02	0.101	0.13 ± 0.09	0.03 ± 0.01	**0.045**
PC P-16:0_20:4	0.13 ± 0.09	0.03 ± 0.02	0.098	0.12 ± 0.09	0.03 ± 0.02	0.101
PC 18:0_20:4	0.56 ± 0.46	0.17 ± 0.12	0.098	0.56 ± 0.41	0.15 ± 0.10	**0.045**
PC 18:1_20:4	0.21 ± 0.14	0.05 ± 0.04	0.098	0.21 ± 0.15	0.03 ± 0.03	**0.039**
PC 18:2_20:4	0.08 ± 0.06	0.02 ± 0.01	0.098	0.09 ± 0.05	0.005 ± 0.003	**0.029**
PC O-18:0_20:4	0.12 ± 0.12	0.05 ± 0.02	0.152	0.11 ± 0.08	0.06 ± 0.04	0.222
PC O-18:1_20:4	0.28 ± 0.23	0.09 ± 0.04	0.165	0.25 ± 0.18	0.11 ± 0.07	0.222
PC 20:1_20:4	0.02 ± 0.01	0.006 ± 0.003	0.101	0.01 ± 0.01	0.006 ± 0.003	0.222
PC 20:2_20:4	0.01 ± 0.009	0.005 ± 0.003	0.152	0.01 ± 0.007	0.005 ± 0.005	0.129
PC 20:3_20:4	0.03 ± 0.01	0.009 ± 0.006	0.098	0.02 ± 0.01	0.009 ± 0.005	0.280
PC 20:4_20:4	0.02 ± 0.01	0.006 ± 0.003	0.098	0.02 ± 0.01	0.003 ± 0.002	**0.039**
LPC 20:4	0.09 ± 0.07	0.06 ± 0.05	0.359	0.07 ± 0.06	0.04 ± 0.01	0.471
LPC						
LPC 16:0	0.13 ± 0.05	0.171 ± 0.12	0.534	0.06 ± 0.03	0.14 ± 0.15	0.434
LPC 16:1	0.002 ± 0.001	0.005 ± 0.003	0.165	0.002 ± 0.0002	0.002 ± 0.001	0.825
LPC 18:0	0.09 ± 0.07	0.06 ± 0.05	0.567	0.07 ± 0.06	0.04 ± 0.01	0.471
LPC 18:1	0.02 ± 0.01	0.02 ± 0.009	0.746	0.01 ± 0.008	0.02 ± 0.02	0.646
LPC 18:2	0.01 ± 0.02	0.01 ± 0.008	0.699	0.01 ± 0.009	0.01 ± 0.01	0.471
LPC 20:3	0.01 ± 0.07	0.005 ± 0.003	0.301	0.009 ± 0.007	0.003 ± 0.002	0.210
PE-DHA						
PE 16:1_22:6	0.04 ± 0.02	0.01 ± 0.006	**0.034**	0.03 ± 0.03	0.02 ± 0.01	0.650
PE 16:0_22:6	0.07 ± 0.03	0.03 ± 0.01	**0.030**	0.06 ± 0.03	0.05 ± 0.03	0.704
PE O-16:0_22:6	0.40 ± 0.28	0.14 ± 0.07	**0.037**	0.34 ± 0.24	0.22 ± 0.15	0.649
PE P-16:0_22:6	0.45 ± 0.29	0.14 ± 0.08	**0.030**	0.41 ± 0.27	0.22 ± 0.16	0.297
PE 18:0_22:6	0.19 ± 0.09	0.08 ± 0.04	**0.030**	0.18 ± 0.10	0.11 ± 0.08	0.274
PE O-18:0_22:6	0.10 ± 0.08	0.02 ± 0.01	**0.037**	0.09 ± 0.09	0.03 ± 0.02	0.560
PE P-18:0_22:6	0.31 ± 0.26	0.05 ± 0.02	**0.030**	0.29 ± 0.28	0.07 ± 0.05	0.234
PE 18:1_22:6	0.11 ± 0.06 (n = 4)	0.06 ± 0.03	0.133	0.10 ± 0.03 (n = 4)	0.09 ± 0.07	0.767
PE 18:2_22:6	0.03 ± 0.01	0.01 ± 0.007	**0.030**	0.03 ± 0.02	0.02 ± 0.01	0.369
LPE 22:6	0.005 ± 0.002	0.003 ± 0.002	0.119	0.005 ± 0.002	0.003 ± 0.002	0.283
PE-ARA						
PE 16:1_20:4	0.26 ± 0.15	0.09 ± 0.04	**0.037**	0.21 ± 0.12	0.09 ± 0.02	0.234
PE 16:0_20:4	1.65 ± 0.95	0.98 ± 0.37	0.120	1.43 ± 0.87	0.80 ± 0.44	0.297
PE O-16:0_20:4	0.50 ± 0.27	0.23 ± 0.09	**0.037**	0.41 ± 0.20	0.21 ± 0.07	0.202
PE P-16:0_20:4	2.42 ± 1.43	1.11 ± 0.45	**0.037**	2.06 ± 1.02	0.97 ± 0.28	0.202
PE 18:0_20:4	2.26 ± 1.19	1.10 ± 0.36	**0.037**	2.03 ± 1.03	0.99 ± 0.34	0.202
PE O-18:0_20:4	0.66 ± 0.45	0.22 ± 0.09	**0.037**	0.59 ± 0.44	0.17 ± 0.04	0.202
PE P-18:0_20:4	2.95 ± 1.93	1.01 ± 0.43	**0.037**	2.67 ± 1.93	0.77 ± 0.17	0.195
PE 18:1_20:4	0.75 ± 0.37	0.36 ± 0.13	**0.037**	0.63 ± 0.27	0.32 ± 0.13	0.202
PE 18:2_20:4	0.18 ± 0.09	0.08 ± 0.03	**0.037**	0.16 ± 0.05	0.07 ± 0.04	0.195
PE 20:4_20:4	0.02 ± 0.009	0.02 ± 0.007	0.114	0.02 ± 0.004	0.01 ± 0.005	0.202
LPE 20:4	0.06 ± 0.08	0.01 ± 0.01	0.093	0.06 ± 0.03	0.002 ± 0.002	0.234

20:4 = ARA, 22:6 = DHA, PC = phosphatidylcholine, PE = phosphatidylethanolamine, LPC = lysophosphatidylcholine, LPE = lysophosphatidylethanolamine. In grey all “O” = ether, “P” = plasmalogen species, nd = not detected. Values are the mean ± SD. In bold, *p* value < 0.05 after FDR calculation

### Levels of PC and PE containing DHA and ARA were reduced in the circulation of male fetus exposed to maternal obesity but not in females

Interestingly, only male fetuses exposed to obesity demonstrated a lower level of phospholipids containing DHA and ARA in umbilical cord venous plasma (Table 2). In particular, we found reduced ester linked PC containing DHA (PC 18:0_22:6, PC 18:1_22:6) by 43 and 51%, ether and plasmalogen linked PE containing DHA (PE O-18:0_22:6, PE P-18:0_22:6) by 55 and 61%, and ARA (PE P-16:0_20:4, PE P-18:0_20:4) by 56 and 58%, respectively. We previously reported reduced PC 16:0_22:6 and LPC 22:6 (LPC-DHA) levels in male fetus of mothers with obesity [20]. The level of ether and plasmalogen PE species containing DHA and ARA remained unchanged in umbilical circulation of female fetuses (Table 2) despite a significant decrease in PE species containing DHA and ARA in female placentas of obese mothers. Similar to the phospholipid analysis in placenta of normal weight subjects, we observed no change in any DHA and ARA PC and PE species in umbilical cord venous plasma when comparing females and males of mothers with normal weight (controls) (Additional file 1: Figure S2).

**Table 2:** Profile of phosphatidylcholine (PC) and phosphatidylethanolamine (PE) containing DHA and ARA in umbilical cord vein from obese (*n* = 16) compared to normal BMI women (*n* = 16) regarding the fetal sex

	Umbilical cord vein					
	Female			Male		
Relative level	Ctr (*n* = 9)	Ob (*n* = 11)	q value (FDR)	Ctr (*n* = 7)	Ob (*n* = 5)	q value (FDR)
PC-DHA						
PC 14:0_22:6	0.02 ± 0.01	0.01 ± 0.008	> 0.999	0.01 ± 0.007	0.007 ± 0.005	0.258
PC 16:0_22:6	2.79 ± 1.10	2.14 ± 0.76	0.724	3.25 ± 0.67	1.86 ± 0.54	**0.046**
PC O-16:0_22:6	0.19 ± 0.08	0.12 ± 0.06	0.675	0.16 ± 0.04	0.17 ± 0.21	0.242
PC P-16:0_22:6	0.08 ± 0.03	0.04 ± 0.02	0.673	0.09 ± 0.03	0.04 ± 0.03	0.057
PC 18:0_22:6	1.87 ± 0.88	1.35 ± 0.50	0.675	1.97 ± 0.23	0.97 ± 0.32	**0.041**
PC 18:1_22:6	0.14 ± 0.05	0.11 ± 0.03	0.675	0.14 ± 0.02	0.10 ± 0.02	0.057
PC O-18:0_22:6	0.17 ± 0.11	0.13 ± 0.05	0.813	0.17 ± 0.06	0.12 ± 0.09	0.363
PC O-18:1_22:6	0.14 ± 0.09	0.09 ± 0.04	0.675	0.12 ± 0.07	0.09 ± 0.09	0.475
LPC 22:6	0.35 ± 0.10	0.30 ± 0.10	0.813	0.39 ± 0.04	0.19 ± 0.05	**0.041**
PC-ARA						
PC 14:0_20:4	0.009 ± 0.002	0.01 ± 0.003	0.813	0.01 ± 0.005	0.01 ± 0.005	0.914
PC 16:0_20:4	9.47 ± 6.90	10.7 ± 2.50	0.813	12.02 ± 3.97	7.03 ± 5.10	0.242
PC 16:1_20:4	0.15 ± 0.17	0.04 ± 0.01	0.831	0.15 ± 0.15	0.06 ± 0.08	0.305
PC O-16:0_20:4	0.89 ± 0.59	1.25 ± 0.39	0.675	1.51 ± 0.73	0.77 ± 0.45	0.216
PC P-16:0_20:4	0.58 ± 0.51	0.67 ± 0.29	0.813	0.95 ± 0.49	0.39 ± 0.24	0.082
PC 18:0_20:4	9.20 ± 6.14	10.3 ± 1.97	0.813	12.4 ± 2.64	6.19 ± 4.29	0.057
PC 18:1_20:4	0.70 ± 0.55	0.82 ± 0.23	0.813	0.96 ± 0.30	0.67 ± 0.22	0.482
PC 18:2_20:4	0.07 ± 0.03	0.07 ± 0.02	0.813	0.10 ± 0.03	0.05 ± 0.02	0.113
PC O-18:0_20:4	1.41 ± 1.19	1.29 ± 0.57	0.813	1.47 ± 0.92	1.25 ± 1.30	0.743
PC O-18:1_20:4	1.32 ± 0.74	1.43 ± 0.61	0.813	1.66 ± 0.89	0.88 ± 0.31	0.299
PC 20:1_20:4	0.07 ± 0.03	0.08 ± 0.02	0.675	0.08 ± 0.03	0.07 ± 0.01	0.951
PC 20:2_20:4	0.04 ± 0.02	0.05 ± 0.02	0.813	0.04 ± 0.02	0.05 ± 0.01	0.840
PC 20:3_20:4	0.05 ± 0.01	0.05 ± 0.01	> 0.999	0.04 ± 0.01	0.04 ± 0.02	0.943
PC 20:4_20:4	0.04 ± 0.02	0.04 ± 0.01	0.813	0.04 ± 0.01	0.02 ± 0.007	0.055
LPC 20:4	9.36 ± 3.51	8.36 ± 2.36	0.813	8.26 ± 1.37	5.97 ± 1.36	0.082
LPC						
LPC 16:0	53.8 ± 25.6	66.9 ± 17.7	0.675	81.4 ± 47.7	51.6 ± 9.2	0.639
LPC 16:1	2.87 ± 1.50	3.51 ± 0.93	0.724	2.49 ± 1.0	2.77 ± 0.45	0.920
LPC 18:0	9.30 ± 5.94	6.41 ± 1.53	0.675	6.65 ± 1.55	5.25 ± 1.78	0.242
LPC 18:1	15.1 ± 5.12	16.5 ± 3.98	0.813	13.7 ± 4.24	10.8 ± 2.19	0.242
LPC 18:2	19.99 ± 8.9	23.14 ± 7.39	0.813	25.8 ± 13.2	13.5 ± 4.22	0.113
LPC 18:3	0.33 ± 0.20	0.31 ± 0.12	> 0.999	0.25 ± 0.03	0.20 ± 0.05	0.159
LPC 20:3	4.16 ± 1.02	4.62 ± 1.55	0.813	4.37 ± 0.96	2.68 ± 0.81	0.055
LPC 20:5	0.30 ± 0.14	0.29 ± 0.14	0.934	0.33 ± 0.10	0.13 ± 0.05	0.055
LPC 22:4	0.20 ± 0.13	0.11 ± 0.03	0.813	0.17 ± 0.06	0.09 ± 0.03	0.057
PE-DHA						
PE 16:1_22:6	0.06 ± 0.03	0.05 ± 0.02	> 0.999	0.06 ± 0.01	0.04 ± 0.01	0.074
PE 16:0_22:6	0.85 ± 0.34	0.95 ± 0.29	0.804	1.26 ± 0.37	0.74 ± 0.33	0.074
PE O-16:0_22:6	0.43 ± 0.21	0.30 ± 0.07	0.456	0.51 ± 0.37	0.30 ± 0.08	0.545
PE P-16:0_22:6	0.81 ± 0.42	0.52 ± 0.23	0.329	0.98 ± 0.59	0.47 ± 0.15	0.074
PE 18:0_22:6	0.52 ± 0.35	0.45 ± 0.21	> 0.999	0.66 ± 0.29	0.35 ± 0.12	0.082
PE O-18:0_22:6	0.07 ± 0.03	0.07 ± 0.02	> 0.999	0.09 ± 0.02	0.04 ± 0.008	**0.024**
PE P-18:0_22:6	0.24 ± 0.14	0.15 ± 0.06	0.329	0.34 ± 0.24	0.13 ± 0.03	**0.048**
PE 18:1_22:6	0.07 ± 0.01	0.08 ± 0.04	> 0.999	0.11 ± 0.03	0.06 ± 0.02	**0.048**
LPE 22:6	0.12 ± 0.05	0.12 ± 0.05	> 0.999	0.14 ± 0.07	0.11 ± 0.03	0.906
PE-ARA						
PE 16:1_20:4	0.14 ± 0.10	0.08 ± 0.03	0.329	0.12 ± 0.06	0.06 ± 0.02	0.232
PE 16:0_20:4	5.29 ± 2.03	6.97 ± 1.84	0.329	6.02 ± 1.45	6.22 ± 2.02	0.805
PE O-16:0_20:4	0.68 ± 0.28	0.68 ± 0.28	> 0.999	0.76 ± 0.23	0.48 ± 0.16	0.082
PE P-16:0_20:4	2.40 ± 1.18	2.12 ± 0.86	> 0.999	2.75 ± 1.08	1.21 ± 0.46	**0.049**
PE 18:0_20:4	4.19 ± 1.46	4.76 ± 1.06	0.771	5.17 ± 1.67	3.82 ± 1.80	0.294
PE O-18:0_20:4	0.56 ± 0.33	0.45 ± 0.18	> 0.999	0.64 ± 0.16	0.35 ± 0.17	0.082
PE P-18:0_20:4	1.83 ± 1.24	1.37 ± 0.51	> 0.999	2.19 ± 0.75	0.90 ± 0.50	**0.048**
PE 18:1_20:4	0.63 ± 0.22	0.74 ± 0.25	> 0.999	0.64 ± 0.19	0.64 ± 0.19	0.957
PE 18:2_20:4	0.04 ± 0.01	nd		0.08 ± 0.07	0.06 ± 0.007	0.945
LPE 20:4	1.24 ± 0.19	1.86 ± 0.57	0.329	1.26 ± 0.29	1.39 ± 0.91	0.892

20:4 = ARA, 22:6 = DHA, PC = phosphatidylcholine, PE = phosphatidylethanolamine, LPC = lysophosphatidylcholine, LPE = lysophosphatidylethanolamine. In grey all “O” = ether, “P” = plasmalogen species, nd = not detected. Values are the mean ± SD. In bold, *p* value < 0.05 after FDR calculation

Conversely to umbilical cord venous plasma, we found decreased sn-1 ester, ether and plasmalogen PC with ARA in umbilical cord arterial plasma of female fetuses exposed to obesity (Additional file 1: Table S4), and no change in those same species in males. Interestingly, LPC 16:0 and LPC 18:2 levels were increased in umbilical artery in the female not in male fetuses in pregnancy complicated by obesity.

### Placental remodeling pathway of phospholipids is differently affected by the fetal sex in pregnancy complicated by obesity

**Fig. 2:**
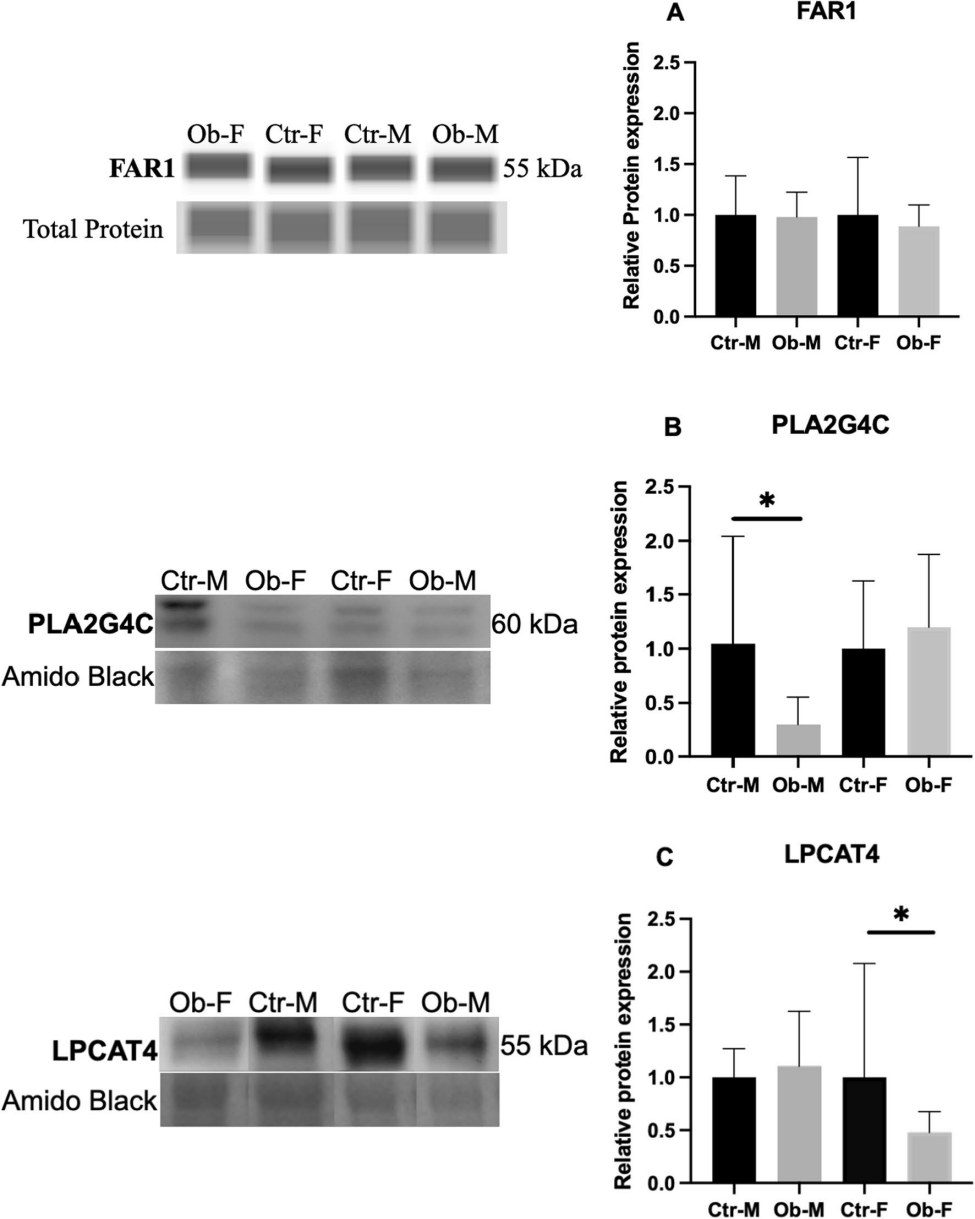
Representative image of capillary electrophoresis bands and relative protein abundance **A** of fatty acyl-CoA reductase 1** (**FAR1) in placental homogenates in obese vs control women in male and female groups obtained by simple West, **B** of PhosphoLipase A2 Group IVC (PLA2G4C or iPLA2) and **C** of LysophosPhatidylCholine Acyl Transferase 4 (LPCAT4). **B** and **C** were analyzed by western blot. Ctr-M = control-Male, Ob-M = Obese-Male, Ctr-F = control-Female, Ob-F = Obese-Female. Data are means ± SD. *p < 0.05

Enzymes involved in the de novo phospholipid synthesis (Kennedy pathway and Lands cycle) were evaluated in placenta homogenates (Additional file 1: Table S2). Additional file 1: Table S5 reports three placental enzymes that were not altered at protein level in association to maternal obesity in male and in female placentas. Relative protein expression of placental Fatty Acyl-CoA Reductase 1 (FAR1), the rate-limiting step for ether lipid biosynthesis in peroxisome is presented in Fig. 2A and was also unchanged in both fetal sexes exposed to maternal obesity.

We also examined the phospholipid remodeling pathway by evaluating expression of PhosphoLipase A_2_ Group IVC (PLA_2_G4C or iPLA_2_) which hydrolyzes acyl groups from PC to form the lysophospholipid (LPC) form and found that PLA_2_G4C abundance was lower in male placentas of obese compared to control mothers (Fig. 2B). We determined the protein expression of LysoPhosphatidylCholine Acyl Transferase 4 (LPCAT4) which catalyzes the re-esterification of acyl chains to LPC to regenerate PC with a unique acyl chain combination and observed a reduction of LPCAT4 abundance in female placentas in pregnancy complicated by obesity (Fig. 2C).

### Relationship between maternal obesity and placental and fetal levels of ether and plasmalogen PE phospholipids

**Fig. 3:**
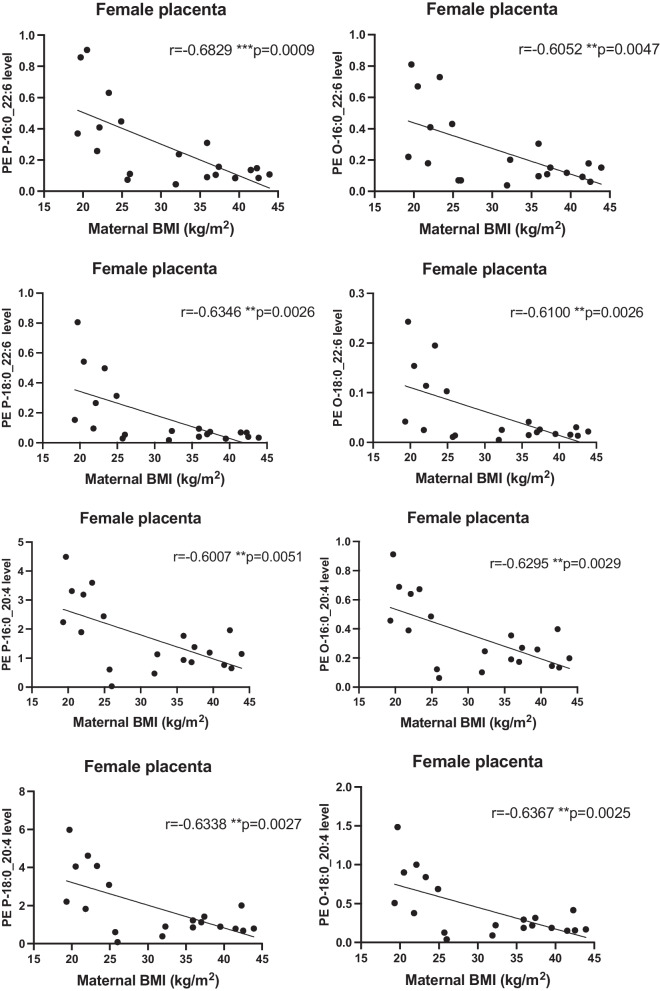
Inverse correlations between maternal BMI and levels of ether and plasmalogen linked PE with DHA and ARA in placentas of female fetus. Spearman test was performed to assess correlations, r and p values are in the figure, *p < 0.05, **p < 0.01, ***p < 0.005

**Fig. 4:**
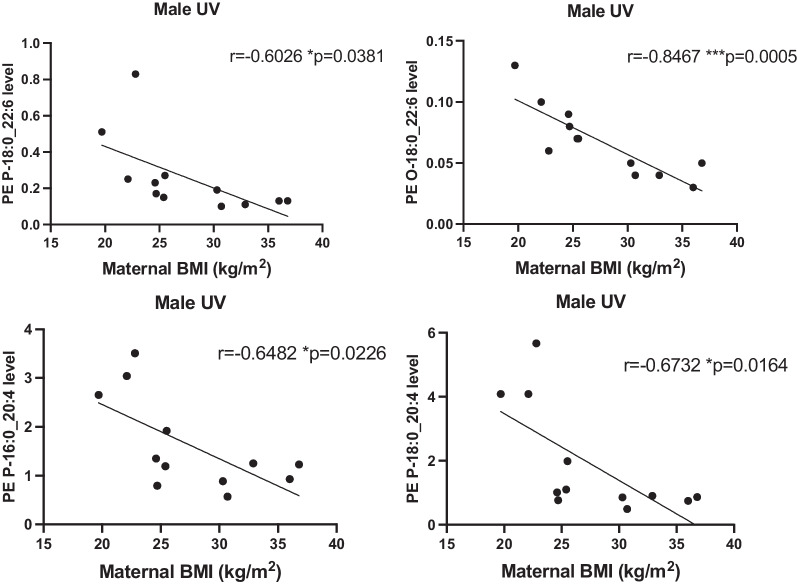
Inverse correlations between maternal BMI and levels of ether and plasmalogen linked PE with DHA and ARA in umbilical cord venous plasma of male fetus. Spearman test was performed to assess correlations, r and p values are in the figure, *p < 0.05, **p < 0.01, ***p < 0.005

In order to examine whether maternal obesity has an incremental impact on placental or cord ether and plasmalogen phospholipids containing DHA or ARA, we tested the relationship between maternal BMI and lipid levels in placenta and umbilical cord. We found an inverse correlation between maternal BMI and the placental tissue levels of the eight ether and plasmalogen PE species containing DHA and ARA in female placentas (Fig. 3). We also found significant inverse correlations between maternal BMI and the levels of four circulating ether and plasmalogen PE species with DHA and ARA in umbilical cord venous plasma of male fetuses (Fig. 4).

**Fig. 5:**
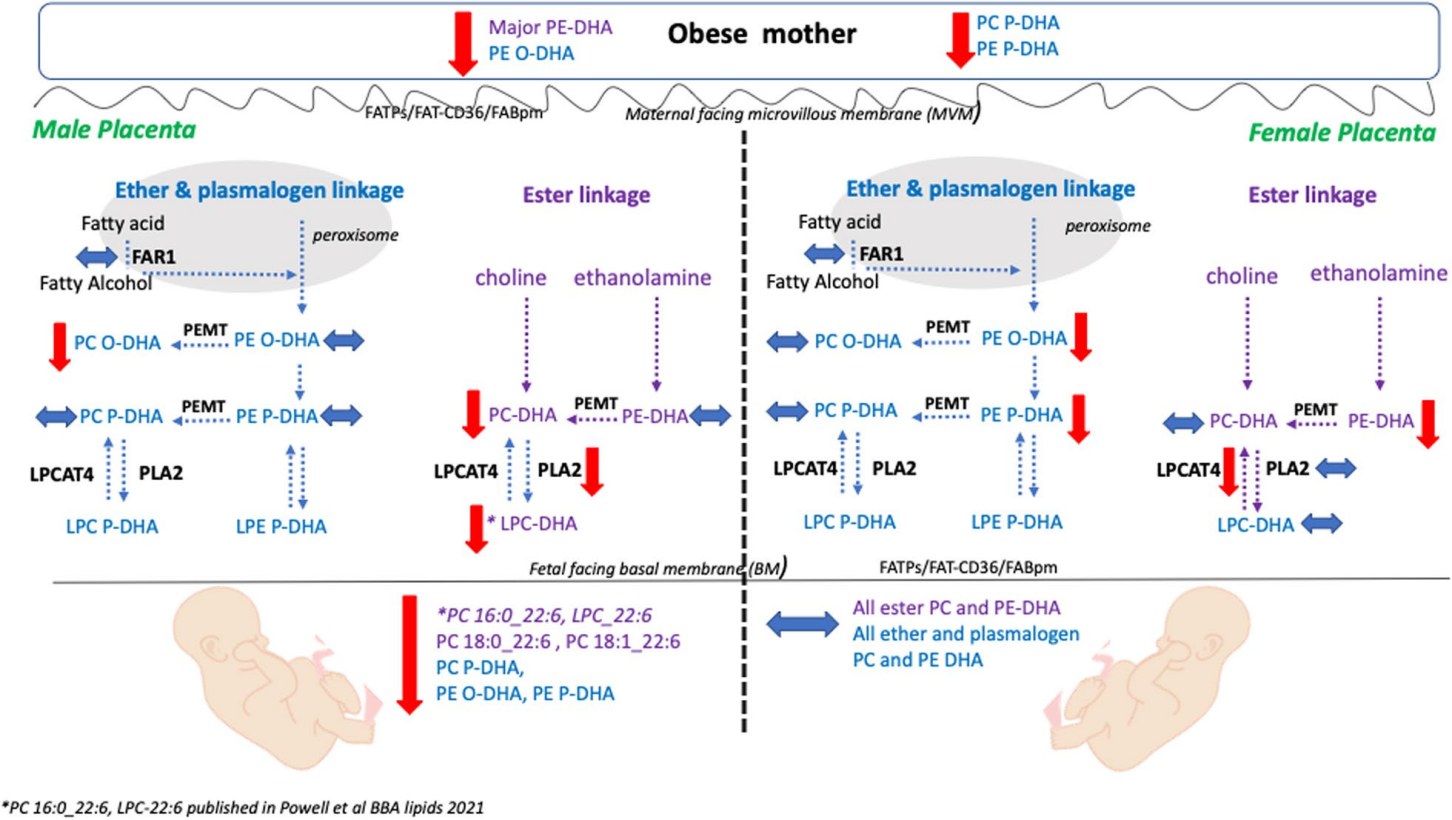
Summary of the influence of fetal sex in ester, ether and plasmalogen PC and PE containing DHA and ARA in the maternal–fetal unit in response to maternal obesity. The phenotype of obese mothers includes reduced ester, ether and plasmalogen PC and PE containing DHA but not those phospholipids containing ARA regardless of fetal sex. In obese mother carrying a male baby: in placenta, all the ester linked PC containing DHA and ARA are decreased by obesity and only one ether PC with DHA species while all ester linked PE were unchanged suggesting downregulated PEMT enzyme activity to convert PE into PC. In addition, decreased LPC-DHA is likely due to decreased iPLA2 protein expression. In fetal circulation, several ester PC DHA and ARA species and ester, ether and plasmalogen PE with DHA and ARA were reduced suggesting a defect of LCPUFA transfer capacity. In obese mother carrying a female baby: in placenta, all PE species with DHA and ARA were decreased but not PC species and LPCAT4 expression was lower. These results suggest an upregulated PEMT activity to maintain PC production. No change was observed in female umbilical cord. By decreasing all ether and plasmalogen PE, species considered as reservoir for DHA and ARA in the placenta, female fetuses of obese mothers show a high fetal–placental adaptability and placental reserve capacity that can maintain the PC-LCPUFA synthesis and the transfer of these crucial species to the fetus to preserve growth and brain development

We provide a summary of the aforementioned results, demonstrating the influence of fetal sex on maternal–placental–fetal adaptation of the DHA and ARA for ester, ether and plasmalogen PC and PE in maternal obesity in Fig. 5.

## Discussion

Our study demonstrates a strong influence of pre-pregnancy maternal BMI on phosphatidylcholine and phosphatidylethanolamine species containing LCPUFA (DHA and ARA) in three compartments: maternal circulation, placenta and fetal umbilical circulation. These changes may have consequences for placental function, birth weight, and fetal brain development as well as lipid signaling regarding the fetal sex. We found significant differences in levels of sn-1 ester, and particularly sn-1 ether and plasmalogen PC and PE containing DHA and ARA in all three compartments in pregnancies complicated by obesity and changes in placenta and cord plasma were highly sex specific. This is the first report to suggest that sn-1 ether and plasmalogen phospholipids containing crucial LCPUFA may play a key role in maternal to fetal transfer by the placenta, and possibly act as a reservoir of vital DHA and ARA for the fetus. We also speculate that the female placenta is able to compensate in the obese environment to more effectively deliver vital lipids to the fetal compartment.

In the maternal circulation of obese women, we found lower levels in several phospholipid species with DHA, including sn-1 ester linked PE 16:0_22:6 and PE 18:0_22:6, sn-1 ether linked PE O-18:0_22:6 and sn-1 plasmalogen linked PC P-16:0_22:6 and PE P-18:0_22:6 but we found no differences in ARA linked phospholipids (Fig. 1). Interestingly, the lower maternal levels of PC and PE species containing DHA were not influenced by fetal sex (Additional file 1: Table S3) and are more likely to be a direct dietary origin. Also, the reduced DHA content in the maternal circulation is related to the obesogenic phenotype and not to the sex of the fetus. These results are in line with our previous study showing reduced DHA in the total circulating phospholipid fraction in plasma of obese women [20]. Lower circulating DHA levels of obese women reflect changes in phospholipid metabolism related to her diet which is often poor in n-3 LCPUFA and high n-6 LCPUFA including ARA. Lower DHA levels and higher n-6 LCPUFA/n-3 LCPUFA ratio have been previously reported in obese pregnant women [41]. In addition, estrogen enhanced the conversion of linolenic acid (ALA, n-3 LCPUFA) into DHA, by increasing the expression of FADS2, the rate-limiting enzyme in the conversion of ALA into DHA, and other transcription factors like PPAR-α, synergically with the low linoleic acid (LA, n-6 LCPUFA) diet [42]. In pregnant obese women, lower estrogen level as well as higher LA diet [41] are described and could explain in part the lower DHA level in circulating phospholipids in the mother.

Among the five PC and PE species containing DHA and decreased in maternal plasma from obese mothers, three are ether and plasmalogen species. Plasmalogens are a subset of ether glycerophospholipids that are characterized by a *cis* double bond adjacent to the ether linkage, forming a vinyl ether linkage. They are considered the most abundant and biologically active class of ether lipids [21, 43]. The distribution of these lipids is variable among tissues with a high content in the brain and paradoxically low levels in liver. The low ether lipid content, including plasmalogens, in the liver may be due to rapid release and incorporation into lipoproteins for transport to other tissues, such as the brain [44]. Several studies have reported that lower ether lipid content in serum is associated with hypertension and obesity [30, 31]. In the context of pregnancy, lower sn-1 ether PC species with DHA suggests reduced DHA enrichment of lipoproteins (HDL, LDL, VLDL) which are essential for lipid transfer across the placenta.

We also observed that maternal obesity affects the placental level of sn-1 ester, ether and plasmalogen PC and PE containing both DHA and ARA but with differential effects of fetal sex (Table 1). In the same cohort, we have previously shown that the protein expression of major fatty acid transporters (FATP1-4, FATP6, FABPM, FAT/CD36) was unchanged in microvillous membrane of the placenta (MVM, maternal facing plasma membrane) in both fetal sexes [20]. Gene (mRNA) expression of placental FATP1 and FATP4 has been correlated with the percent of phospholipids containing DHA in maternal plasma in normal pregnancy indicating a key role of those transporters for uptake of DHA from the mother [45]. However, these fatty acid transporters can also transport other fatty acids such as the saturated and monounsaturated fatty acids (SFA and MUFA). With lower DHA in circulation of obese mothers, the action of these transporters in the placenta is likely to take up additional SFA or MUFA or n-6 LCPUFA all of which are highly abundant in maternal plasma. In our study, a decrease of DHA esterification into PC (PC 16:0_22:6, PC 18:0_22:6 for males) and PE (PE 16:0_22:6, PE 16:1_22:6, PE 18:0_22:6, PE 18:2_22:6 and similar PE species with ether and plasmalogen linkages for females) (Table 1) in the placental phospholipid fraction could, in part, be explained by lower DHA substrate availability rather than a deficient transport capacity in MVM for uptake of DHA by both male and female placentas [46]. However, obese mothers with both male and female fetuses had lower DHA content in their plasma but placental phospholipid species levels were distinct in the sexes suggesting that metabolism and transfer of DHA differs by fetal sex and that maternal supply is not the only parameter of interest. This suggestion is also valid for ARA which is not altered in maternal plasma by obesity but is differentially reduced in placental PC and PE when analyzing the data based on fetal sex (Table 1). Decreased placental PC and PE with DHA and ARA in pregnancies complicated by maternal obesity may be due to increased phospholipid synthesis with SFA and MUFA at the sn-2 position to maintain phospholipid content in the expanding exchange surface area of the placenta across gestation. That process may take precedence over delivery of LCPUFA to the fetus when DHA is not readily available. In this study, we did not quantify the PC and PE species with specific SFA and/or MUFA in both sn-1 and sn-2 positions, therefore we provide this as a speculation for how placentas might respond to low DHA availability. Placental sex differences in PC and PE containing DHA and ARA were not found in pregnancies with normal weight mothers (Additional file 1: Figure S1) suggesting the findings in this report are a specific response to maternal obesity that differs by fetal sex.

Another possible explanation for the placental decreases in preferred forms of PC and PE containing LCPUFA could be that other lipid complexes such are triacylglycerols (TG), cellular storage form, are synthesized at the expense of incorporation of DHA and ARA into phospholipids. In our previous study, we have shown that DHA and ARA levels in total TG remained stable in both male and female placentas of obese women while the non-esterified form of both FAs were decreased along with a trend to be decreased in total phospholipid pool only in male placenta. This indicates a possible preferential mobilization of DHA and ARA toward the triglyceride form [20]. Since obesity during pregnancy is associated with placental inflammation and hypoxic stress [47], increased storage of LCPUFA in TG may be similar to what has been previously reported for intrauterine growth restriction (IUGR) placentas [48, 49].

Interestingly, we show a specific decrease of particular sn-1 ether and plasmalogen PE species with DHA and ARA in placentas from obese mothers carrying female fetuses (Table 1). Since ether and plasmalogen lipids are specifically synthesized in the peroxisome [43], lower levels in the female placentas of obese women in our study may suggest a defect of peroxisomal ether linked phospholipid production. Peroxisomes are membrane bound organelles that perform multiple functions, including ether lipid synthesis and fatty acid oxidation [50]. In the same cohort, we have previously shown a down-regulation of beta-oxidation in female placentas of obese women suggesting a mitochondrial dysfunction and perhaps a peroxisome deficiency [20]. Mitochondrial dysfunction has been observed in trophoblast cells of female placentas in response to maternal obesity resulting from an activation of signaling from inflammation via NFκB1 and miR-210 [51]. Peroxisomal dysfunction is characterized by reduced levels of plasmalogens contributing to various metabolic pathologies such as dyslipidemia, obesity, NAFLD and T2D possibly through multiple pathways, including the disruption of cellular membranes, increased oxidative stress, endoplasmic reticulum (ER) stress and inflammation [50].

The production of fatty alcohols from fatty acyl-CoA is needed for generation of the ether bond and is mediated by FAR1. This has been proposed as rate-limiting step for ether lipid biosynthesis in the peroxisome [52]. Substrate availability and activity of FAR1 are essential regulators of the entire ether phospholipid synthesis pathway [53]. FAR1 accounting for C16:0, C18:0, and C18:1 fatty alcohol synthesis is post-translationally regulated by the accumulation of plasmalogen PE in the inner leaflet of the plasma membrane [54]. In our study, placental FAR1 protein expression was not modified by maternal obesity in either fetal sex (Fig. 2A) while the placental plasma membrane levels of plasmalogen PE containing DHA and ARA were decreased. Since DHA and ARA are transferred to PE during the remodeling pathway (land’s cycle), FAR1 activity is likely upstream regulated by PE containing other FAs in sn-2 position than DHA or ARA.

Two other peroxisomal enzymes play an important role in the initiation of the ether lipid synthesis; the glyceronephosphate O-acyltransferase (GNPAT), that acylates dihydroxyacetone phosphate (DHAP) with a fatty acyl-CoA at the sn-1 position and the alkylglycerone phosphate synthase (AGPS) that catalyzes the exchange of the fatty acyl group of 1-acyl-DHAP for a fatty alcohol [43]. However, we were not able to quantify the expression of those enzymes at the protein level in the placenta due to lack of specific antibodies.

Despite the unchanged expression in FAR1 enzyme, lower DHA supplied in the plasma of obese mothers may alter the ether and plasmalogen PE and PC synthesis outside the peroxisome. Indeed, phospholipid synthesis is completed at the ER mediated by specific enzymes involved in the de novo Kennedy pathway and remodeling pathways (Land’s cycle) [43]. We found lower levels of sn-1 ether and plasmalogen linked PE with DHA and ARA, and no change in sn-1 ether and plasmalogen PC with DHA and ARA in female placentas of obese women suggesting an enhanced phosphatidylethanolamine methyl transferase (PEMT) enzyme activity to maintain de novo synthesis of ether and plasmalogen PC in the ER. This may act as a reservoir of DHA and ARA to be transferred to the fetus through generation of PC species which can be transporters as LPC through the MFSD2a transporter in the basal plasma membrane. This placental response to obesity may be a compensatory mechanism in response to deficient phospholipid remodeling pathway as indicated by the decreased expression of LPCAT4 in female placentas (Fig. 2C). Furthermore, the unchanged levels of PC and PE containing DHA and ARA in the umbilical cord venous plasma in pregnancies carrying female fetuses (Table 2) as well as our previous study showing that LPC-DHA was not changed in female placentas and in female fetal umbilical circulation exposed to obesity [20] is consistent with the concept that the fetus and placenta have the ability to adapt to preserve the transfer capacity of LCPUFA to the developing female fetus. We observed an inverse correlation between the placental level of sn-1 ether and plasmalogen PE containing DHA and ARA species and maternal BMI (Fig. 3) combined with altered expression of LPCAT4 enzyme, which potentially reduces the re-esterification of those PE species through incorporation of a LCPUFA into the sn-2 position of LPC. This complexity suggests a possible interplay between peroxisome derived ether phospholipids and the specific enzymes involved in the phospholipid remodeling pathway in placentas of obese mothers carrying a female fetus. We have attempted to summarize these findings in Fig. 5 for clarity.

In obese pregnant mothers carrying males, we observed levels of sn-1 ester linked PC with DHA and ARA profoundly reduced in placenta, and no change in PE forms (Table 1). The lower PC DHA and ARA level in males is likely dependent on the PEMT pathway that converts PE into PC. While unable to quantify PEMT protein expression in the placenta, we speculate that the PEMT enzyme activity is downregulated in male placentas exposed to maternal obesity. Moreover, lower levels of major PC-DHA species (PC 16:0_22:6, PC 18:0_22:6) likely lead to decreased LPC-DHA in male placentas of obese mothers as we have recently reported [20]. This may be indirectly mediated by decreased PLA_2_G4C (iPLA2) protein expression in male but not in female placentas (Fig. 2B). Indeed, iPLA_2_ cleaves at the sn-2 position of PC and preferentially released LCPUFA. Less DHA released in the remodeling phospholipid pathway may be reflected in decreased non-esterified DHA [20]. In addition, lower levels of PC containing DHA species in placenta may cause a defect of choline and DHA released and transferred to the male fetus with potential consequences for the brain development, since choline promotes the DHA transport as LPC through the blood brain barrier mediated by the mammalian family super domain 2a, MSFD2a transporter [25].

Lower circulating choline may be related to the observation of a reduced levels of ether and plasmalogen PC and PE containing DHA and ARA in the fetal circulation of males of obese mothers. Indeed, choline is a substrate of all forms of PC synthesis, and in case of ester PC linkage, can be converted into sphingomyelin (SM) metabolites including SM containing very long chain fatty acid, such as nervonic acid (24:1). This fatty acid is exclusively catabolized in the peroxisome and its breakdown is considered a source for alkyl groups in plasmalogen synthesis [55]. Inversely, a supplementation of choline to pregnant mice fed with a high-fat diet increases the relative abundance of hepatic plasmalogen and sphingomyelin d42:2 (SM d18:1_24:1, containing nervonic acid) in fetus at 17.5 day of gestation and after 6 weeks postnatal in male offspring, indicating an antioxidative response to protect the mouse liver from damages due to high-fat feeding [56]. Another study has shown that choline supplementation increased the incorporation of DHA into choline-containing phospholipids in a mouse model of maternal obesity [57].

Lower levels of ether and plasmalogen phospholipids at birth in male infants (Table 2) may be a signature for higher risk to develop metabolic diseases later in life. In children diagnosed with type 1 diabetes, reduced ether lipids (such as PE O-18:1_20:4) were detected in the serum prior to the detection of autoantibodies [58]. Male fetuses have higher nutritional requirements as evidenced by studies observing that women carrying male babies consume more nutrients, especially lipids [59]. Thus, male fetal growth is particularly sensitive to maternal dietary content and a diet deficient in n-3 LCPUFA in obese mothers appears to more profoundly affect male rather than female fetuses with respect to circulating DHA in the umbilical circulation.

Although the effect of decreased DHA supply on brain growth and development remains to be fully established, we speculate that altered placental PC-DHA metabolism and less DHA availability in circulation of male fetuses exposed to obesity may negatively influence the development of the brain, programming early life neurodevelopmental disorders. Indeed, dietary n-3 fatty acid deficiency reduces DHA-regulated PLA_2_ and calcium-independent iPLA_2_ in the frontal cortex in rodents and subsequently downregulates the brain phospholipid remodeling pathway [60].

The mechanisms that drive the sex-specific adaptations that we show in the placental phospholipid metabolism including the ether linked PC and PE containing DHA and ARA in the setting of obesity (Fig. 5) remain largely unexplored. Literature evidences suggest that the feto-placental hormonal environment could play a major contributor. An important role of androgens was described in regulation of fetal growth and development [61] and changes in the placental–fetal hormonal environment due to maternal obesity may contribute to sex-specific differences in placental lipid metabolism. Further studies need to be developed to determine the hormonal effect on ether lipid in placenta and in fetal circulation in both sexes in normal pregnancy and in pregnancy complicated by obesity.

Limitations of this study include the small number of enrolled women and smaller sample size in each fetal sex group and lack of dietary information from the pregnant women. In addition, we were not able to find acceptable antibodies for many peroxisome and lipid synthesis enzymes involved in ether and plasmalogen synthesis. One of the major strengths of the study is that we have specifically identified the plasmalogen and ether linked phospholipids and analyzed those species in maternal and umbilical cord plasma as well as placentas of healthy mothers and those complicated by obesity and that those maternal–placental–fetal samples provide from the same pregnancy.

### Perspectives and significance

We show that maternal obesity reduces the content of placental PC and PE containing LCPUFA differentially in women carrying male compared to female infants. This difference has potential consequences on supply of this preferred lipid form for fetal brain development with short- and long-term consequences. This study is the first to explore the two important phospholipids; PC and PE containing DHA and ARA, and examined sn-1 ester, ether and plasmalogen linkages, in maternal and fetal circulation and in placental tissue to uncover potential novel roles for ether and plasmalogen lipids in the regulation of placenta delivery of these vital nutrients in pregnancies complicated by obesity. By decreasing all ether and plasmalogen PE, species which are considered a reservoir for DHA and ARA in the placenta, female fetuses of obese mothers show a high fetal–placental adaptability and placental reserve capacity that can maintain the PC-LCPUFA (including LPC-DHA) synthesis and the transfer of these crucial species to the fetus to preserve growth and brain development. Male fetuses, in response to maternal obesity, reduce the placental ester PC species containing DHA and ARA as well as reduce the ether and plasmalogen PE reservoir of DHA and ARA in fetal circulation. Both males and females appear to have compensatory mechanisms that attempt to maintain an adequate supply of LCPUFA species for fetal organ growth, in particular the brain. The adaptation in females maintains normal levels of DHA and ARA in the umbilical circulation while lower plasmalogen PE LCPUFA contents in the male umbilical circulation at birth appears to be a failure of the adaptive responses. This could explain, in part, the male fetus being more vulnerable to the long-term detrimental consequences of gestation in the environment of maternal obesity. Further molecular studies will help to better understand the mechanism of ether and plasmalogen phospholipids in maternal–fetal unit and the role of fetal sex.

### Supplementary Information

**Additional file 1. **Supplemental data.

## Data Availability

All the data that support the findings are presented in the manuscript and in the supplementary data.
