# Supplementary material for: Fetal sex differences in placental LCPUFA ether and plasmalogen phosphatidylethanolamine and phosphatidylcholine contents in pregnancies complicated by obesity
Source: Biol Sex Differ. 2023 Sep 28;14:66. doi: 10.1186/s13293-023-00548-1 (PMC10540428; doi:10.1186/s13293-023-00548-1)
Supplement: Supplementary file 1 — Additional file 1. Supplemental data. [file 13293_2023_548_MOESM1_ESM.pdf]

Supplemental data

Table S1A: Clinical parameters of mothers and infants

Variable	Control	Obese
**N**	16	16
Maternal BMI (kg/m^2)	23.0 ± 2.3	36.7 ± 4.3*
Age at delivery (years)	31.4 ± 5.0	29.4 ± 5.5
Gestational age (weeks)	39.1 ± 1.1	39.3 ± 0.6
Delivery mode (C/V)	12/4	16/0
Placental weight (g)	647.2 ± 116	705.7 ± 222
Birth weight (g)	3369.9 ± 379	3608.9 ± 763
Infant Sex	7 males/9 females	5 males/11 females

Data are presented as mean ± SD. *p value (t-test) < 0.05 C/S=Cesarean Section

Table S1B: Mothers and infants clinical parameters separated in fetal sex.

	Female		Male	
**Variable**	**Control**	**Obese**	**Control**	**Obese**
Plasma and placenta of triads (mother-placenta-fetus) used for phospholipid quantification				
**N**	9	11	7	5
Maternal BMI (kg/m^2)	22.5 ± 2.4	38.2 ± 4.1***	23.5 ± 2.1	33.3 ± 3.0**
Age at delivery (years)	31.0 ± 4.1	30.0 ± 5.1	31.9 ± 6.4	28.0 ± 6.6
Gestational age (weeks)	39.1 ± 1.1	39.2 ± 0.2	39.3 ± 1.2	39.6 ± 1.1
Delivery mode (C/V)	7/2	11/0	5/2	10/0
Placental weight (g)	650.4 ± 112	732.5 ± 198	643.2 ± 129	646.9 ± 286
Birth weight (g)	3323.3 ± 466	3559.1 ± 489	3429.9 ± 250	3718.4 ± 1250
Placental tissue used for western blotting or Jess study				
**N**	10	10	8	10
Maternal BMI (kg/m^2)	22.6 ± 0.8	38.4 ± 4.2***	23.0 ± 2.5	36.6 ± 4.8 ***
Age at delivery (years)	30.3 ± 4.5	30.2 ± 5.3	32.6 ± 6.3	29.6 ± 5.8
Gestational age (weeks)	39.2 ± 1.2	39.1 ± 0.1	39.2 ± 1.1	39.4 ± 0.8
Delivery mode (C/V)	8/2	10/0	6/2	10/0
Placental weight (g)	634.1 ± 126	726.7 ± 207	634.1 ± 123	660.1 ± 240
Birth weight (g)	3282.0 ± 497	3507.0 ± 482	3397.1 ± 250	3696.4 ± 865

Data are presented as mean ± SD. **p value < 0.005 (t-test) Control vs Obese, ***p value < 0.001, C = Caesarean section, V = vaginal delivery.

Table S2: Information of Antibodies used in JESS and Western Blot (WB).

Peptide/Protein Target	Name of Antibody	Species raised in Monoclonal or Polyclonal	Manufacturer and Catalog No.	Protein Assay	Dilution Used
**AGPAT2**	Anti-AGPAT2	Rabbit;Polyclonal	Sigma HPA019544	JESS	1:100
**GPAT3**	Anti-GPAT3	Rabbit;Polyclonal	Sigma HPA029414	JESS	1:100
**LPCAT4**	Anti-LPCAT4	Rabbit;Polyclonal	Invitrogen PA5-50544	WB	1:1000
**PLA2G4C**	Anti-PLA2G4C	Rabbit;Polyclonal	Sigma HPA043083	WB	1:1000
**FAR1**	Anti-FAR1	Rabbit;Polyclonal	Novus NBP1-89847	JESS	1:200
**AGPAT4**	Anti-AGPAT4	Rabbit;Polyclonal	Novus NBP1-79870	WB	1:1000

Table S3: Profile of phosphatidylcholine (PC) and phosphatidylethanolamine (PE) containing DHA and ARA in maternal circulation of normal BMI (n=16) compared to obese women (n=16) regarding the fetal sex.

	Maternal Plasma					
	**Female**			**Male**		
Relative level	**Ctr (n=9)**	**Ob (n=11)**	**p value (FDR)**	**Ctr (n=7)**	**Ob (n=5)**	**p value (FDR)**
PC-DHA						
PC 14:0_22:6	0.15 ± 0.13	0.13 ± 0.02	0.695	0.18 ± 0.11	0.10 ± 0.02	0.623
PC 16:0_22:6	10.89 ± 4.60	8.18 ± 2.85	0.393	12.47 ± 4.59	7.47 ± 2.94	0.273
PC O-16:0_22:6	0.96 ± 0.31	0.68 ± 0.21	0.260	1.36 ± 0.48	0.98 ± 0.38	0.553
PC P-16:0_22:6	0.48 ± 0.23	0.26 ± 0.10	0.142	0.60 ± 0.23	0.35 ± 0.12	0.273
PC 18:0_22:6	6.65 ± 3.81	3.89 ± 1.61	0.342	8.05 ± 4.49	5.39 ± 1.19	0.356
PC 18:1_22:6	1.14 ± 0.61	0.62 ± 0.29	0.260	1.01 ± 0.50	0.73 ± 0.21	0.623
PC O-18:0_22:6	1.17 ± 0.50	0.75 ± 0.46	0.273	1.80 ± 1.10	1.21 ± 0.50	0.691
PC O-18:1_22:6	1.32 ± 0.48	0.85 ± 0.35	0.260	1.79 ± 0.86	1.18 ± 0.45	0.522
LPC_22:6	0.09 ± 0.06	0.06 ± 0.04	0.443	0.11 ± 0.04	0.07 ± 0.03	0.230
PC-ARA						
PC 14:0_20:4	0.03 ± 0.02	0.02 ± 0.01	0.695	0.04 ± 0.03	0.06 ± 0.03	0.509
PC 16:0_20:4	22.9 ± 16.8	29.1 ± 18.9	0.804	36.8 ± 20.3	44.2 ± 15.3	0.691
PC 16:1_20:4	0.45 ± 0.39	0.04 ± 0.02	0.443	0.40 ± 0.48	0.05 ± 0.03	0.806
PC O-16:0_20:4	4.50 ± 3.40	2.08 ± 2.97	0.542	4.22 ± 3.59	7.02 ± 2.36	0.623
PC P-16:0_20:4	3.99 ± 1.07	1.13 ± 0.59	0.260	3.63 ± 1.16	4.18 ± 4.73	0.806
PC 18:0_20:4	22.7 ± 18.5	8.63 ± 13.9	0.342	24.14 ± 23.4	26.30 ± 20.3	0.906
PC 18:1_20:4	3.60 ± 2.94	2.23 ± 2.47	0.870	3.86 ± 3.04	5.29 ± 1.95	0.770
PC 18:2_20:4	0.48 ± 0.27	0.49 ± 0.29	>0.999	0.79 ± 0.28	0.80 ± 0.22	0.991
PC O-18:0_20:4	6.73 ± 2.54	5.10 ± 2.95	0.260	8.25 ± 2.99	5.94 ± 4.41	0.623
PC O-18:1_20:4	9.07 ± 4.13	5.62 ± 3.70	0.262	9.77 ± 4.51	8.90 ± 7.39	0.981
PC 20:1_20:4	0.64 ± 0.33	0.35 ± 0.37	0.273	0.54 ± 0.37	0.52 ± 0.23	>0.999
PC 20:2_20:4	0.17 ± 0.09	0.16 ± 0.15	0.870	0.20 ± 0.13	0.22 ± 0.06	0.806
PC 20:3_20:4	0.10 ± 0.03	0.09 ± 0.06	>0.999	0.12 ± 0.07	0.17 ± 0.07	0.623
PC 20:4_20:4	0.09 ± 0.04	0.12 ± 0.05	0.870	0.15 ± 0.05	0.11 ± 0.05	0.742
LPC_20:4	0.79 ± 0.36	0.80 ± 0.24	0.870	0.98 ± 0.38	0.89 ± 0.23	0.981
LPC						
LPC_16:0	37.7 ± 18.8	31.7 ± 14.2	0.842	38.3 ± 15.28	22.3 ± 7.85	0.167
LPC_16:1	0.66 ± 0.37	0.71 ± 0.32	0.870	0.81 ± 0.63	0.47 ± 0.16	0.785
LPC_18:0	10.21 ± 3.53	8.69 ± 3.85	0.443	11.03 ± 3.21	5.71 ± 2.09	0.148
LPC_18:1	6.16 ± 1.62	4.98 ± 2.20	0.366	7.10 ± 2.04	3.60 ± 0.98	0.085
LPC_18:2	6.84 ± 3.64	5.97 ± 2.48	0.758	9.66 ± 3.60	5.62 ± 2.11	0.167
LPC_18:3	0.14 ± 0.08	0.14 ± 0.03	0.695	0.21 ± 0.11	0.13 ± 0.05	0.384
LPC_20:3	0.51 ± 0.16	0.53 ± 0.19	>0.999	0.85 ± 0.39	0.42 ± 0.16	0.085
LPC_22:4	0.03 ± 0.01	0.04 ± 0.01	0.956	0.06 ± 0.02	0.02 ± 0.01	0.148
PE-DHA						

PE 16:1_22:6	0.22 ± 0.14	0.42 ± 0.93	0.696	0.22 ± 0.10	0.15 ± 0.04	0.593
PE 16:0_22:6	6.84 ± 4.55	4.08 ± 1.68	0.305	7.16 ± 4.05	3.86 ± 0.41	0.176
PE O-16:0_22:6	1.31 ± 0.73	0.74 ± 0.20	0.305	1.63 ± 1.19	1.02 ± 0.28	0.886
PE P-16:0_22:6	2.00 ± 1.21	1.17 ± 0.35	0.307	2.48 ± 2.05	1.38 ± 0.56	0.653
PE 18:0_22:6	3.85 ± 2.97	1.88 ± 0.86	0.267	4.53 ± 3.87	1.89 ± 0.34	0.503
PE O-18:0_22:6	0.45 ± 0.29	0.18 ± 0.08	0.267	0.50 ± 0.35	0.20 ± 0.07	0.327
PE P-18:0_22:6	1.36 ± 1.09	0.44 ± 0.15	0.267	1.72 ± 1.13	0.52 ± 0.16	0.327
PE 18:1_22:6	0.90 ± 0.21	0.67 ± 0.24	0.307	0.54 ± 0.10	0.60 ± 0.16	0.938
LPE_22:6	0.04 ± 0.02	0.04 ± 0.03	0.980	0.06 ± 0.02	0.03 ± 0.008	**0.025**
PE-ARA						
PE 14:0_20:4	0.11 ± 0.08	0.09 ± 0.02	0.980	0.15 ± 0.06	0.14 ± 0.02	0.938
PE 16:1_20:4	0.77 ± 0.93	0.26 ± 0.09	0.335	0.81 ± 0.68	0.17 ± 0.11	0.327
PE 16:0_20:4	24.0 ± 12.4	28.4 ± 7.4	0.696	31.3 ± 12.9	22.5 ± 10.6	0.504
PE O-16:0_20:4	2.21 ± 1.26	1.68 ± 0.43	0.980	2.84 ± 1.31	2.02 ± 0.71	0.653
PE P-16:0_20:4	6.78 ± 3.50	5.13 ± 1.33	0.647	9.09 ± 4.0	6.78 ± 2.65	0.850
PE 18:0_20:4	17.9 ± 3.0	15.3 ± 1.3	0.867	22.6 ± 14.2	15.2 ± 5.1	0.938
PE O-18:0_20:4	3.43 ± 2.74	1.63 ± 0.28	0.305	4.37 ± 2.96	2.13 ± 0.71	0.653
PE P-18:0_20:4	10.4 ± 7.38	5.00 ± 0.88	0.305	14.76 ± 9.53	6.88 ± 2.17	0.653
PE 18:1_20:4	4.69 ± 2.22	3.90 ± 1.04	0.502	5.46 ± 2.99	3.68 ± 1.42	0.886
PE 18:2_20:4	0.27 ± 0.10	0.19 ± 0.08	0.305	0.33 ± 0.10	0.25 ± 0.12	0.653
LPE_20:4	0.35 ± 0.13	0.34 ± 0.08	0.980	0.57 ± 0.28	0.35 ± 0.07	0.282

20:4 = ARA, 22:6 = DHA, PC = phosphatidylcholine, PE = phosphatidylethanolamine

LPC = Lysophosphtidylcholine, LPE = lysophosphatidylethanolamine

In grey all "O" = ether, "p" = plasmalogen species

Values are the mean ± SD. In bold p value < 0.05 after FDR calculation.

Table S4: Profile of PC and PE containing DHA and ARA in umbilical cord artery from normal BMI (n=16) compared to obese women (n=16) regarding the fetal sex.

	**Umbilical cord artery**					
	**Female**			**Male**		
Relative Level	**Ctr (n=9)**	**Ob (n=11)**	**p value (FDR)**	**Ctr (n=7)**	**Ob (n=5)**	**p value (FDR)**
PC-DHA						
PC 14:0_22:6	0.02 ± 0.01	nd		nd	nd	
PC 16:0_22:6	3.51 ± 2.73	1.92 ± 0.81	0.089	2.77 ± 0.38	1.25 ± 0.33	0.060
PC O-16:0_22:6	0.28 ± 0.31	0.18 ± 0.11	0.796	0.15 ± 0.05	0.15 ± 0.12	0.901
PC P-16:0_22:6	0.08 ± 0.06	0.08 ± 0.05	0.575	0.07 ± 0.02	0.05 ± 0.03	0.712
PC 18:0_22:6	2.09 ± 1.44	1.42 ± 0.63	0.266	1.53 ± 0.28	1.54 ± 1.45	0.481
PC 18:1_22:6	0.15 ± 0.07	0.09 ± 0.04	0.188	0.11 ± 0.02	0.10 ± 0.02	0.616
PC O-18:0_22:6	0.22 ± 0.27	0.17 ± 0.08	0.353	0.11 ± 0.05	0.17 ± 0.12	0.691
PC O-18:1_22:6	0.19 ± 0.26	0.13 ± 0.07	0.790	0.09 ± 0.03	0.12 ± 0.10	>0.999
LPC_22:6	0.32 ± 0.06	0.24 ± 0.03	0.386	0.35 ± 0.11	0.17 ± 0.06	0.059
PC-ARA						
PC 14:0_20:4	0.02 ± 0.005	0.01 ± 0.006	0.188	0.01 ± 0.003	0.007 ± 0.006	0.551
PC 16:0_20:4	8.73 ± 4.36	0.18 ± 0.13	**0.017**	7.09 ± 6.39	0.21 ± 0.18	0.455
PC 16:1_20:4	0.20 ± 0.12	nd		0.21 ± 0.14	nd	
PC O-16:0_20:4	0.78 ± 0.33	0.15 ± 0.10	**0.022**	0.65 ± 0.53	0.30 ± 0.17	0.848
PC P-16:0_20:4	0.63 ± 0.33	0.08 ± 0.07	**0.022**	0.49 ± 0.49	0.07 ± 0.06	0.455
PC 18:0_20:4	6.24 ± 4.15	0.12 ± 0.18	**0.022**	5.37 ± 4.87	0.47 ± 0.61	0.303
PC 18:1_20:4	0.80 ± 0.25	nd		0.90 ± 0.19	nd	
PC 18:2_20:4	0.07 ± 0.02	nd		0.08 ± 0.01	nd	
PC O-18:0_20:4	0.97 ± 0.64	1.66 ± 0.79	**0.048**	0.68 ± 0.23	1.44 ± 0.97	0.381
PC O-18:1_20:4	1.31 ± 0.88	1.19 ± 0.67	0.790	0.78 ± 0.32	1.24 ± 0.57	0.481
PC 20:1_20:4	0.06 ± 0.03	0.05 ± 0.03	0.575	0.04 ± 0.02	0.05 ± 0.03	0.822
PC 20:2_20:4	0.05 ± 0.04	nd		0.03 ± 0.01	nd	
PC 20:3_20:4	0.03 ± 0.01	nd		0.04 ± 0.02	nd	
PC 20:4_20:4	0.03 ± 0.02	Nd		0.04 ± 0.02	Nd	
LPC_20:4	6.84 ± 2.13	8.93 ± 4.41	0.364	6.78 ± 1.33	5.07 ± 1.69	0.381
LPC						
LPC_16:0	52.4 ± 23.1	112.3 ± 40.4	**0.017**	50.9 ± 19.6	103.5 ± 50.1	0.173
LPC_16:1	1.89 ± 0.81	2.16 ± 0.84	0.455	0.82 ± 0.59	1.94 ± 0.53	0.173
LPC_18:0	6.75 ± 2.45	4.05 ± 1.07	0.066	6.50 ± 1.38	3.18 ± 0.57	0.072
LPC_18:1	12.9 ± 3.97	14.6 ± 3.36	0.302	11.53 ± 2.6	9.95 ± 3.6	0.616
LPC_18:2	16.3 ± 5.45	28.6 ± 9.49	**0.022**	18.80 ± 8.0	18.74 ± 5.7	0.919
LPC_18:3	0.28 ± 0.06	0.26 ± 0.10	0.575	0.18 ± 0.10	0.15 ± 0.02	0.551
LPC_20:3	4.04 ± 1.74	5.12 ± 1.76	0.322	4.40 ± 0.85	3.12 ± 0.79	0.371
LPC_20:5	0.29 ± 0.10	0.33 ± 0.17	0.621	0.24 ± 0.09	0.15 ± 0.05	0.455
LPC_22:4	0.13 ± 0.05	0.20 ± 0.12	0.302	0.19 ± 0.09	0.15 ± 0.07	0.551
PE-DHA						
PE 16:1_22:6	0.05 ± 0.01	0.05 ± 0.01	>0.999	0.06 ± 0.02	0.02 ± 0.01	0.169
PE 16:0_22:6	0.73 ± 0.21	0.84 ± 0.35	0.895	1.11 ± 0.27	0.63 ± 0.20	0.082
PE O-16:0_22:6	0.39 ± 0.12	0.23 ± 0.11	0.146	0.39 ± 0.19	0.24 ± 0.07	0.182
PE P-16:0_22:6	0.80 ± 0.27	0.46 ± 0.25	0.146	0.77 ± 0.35	0.37 ± 0.15	0.140
PE 18:0_22:6	0.41 ± 0.21	0.35 ± 0.19	0.735	0.55 ± 0.14	0.29 ± 0.12	0.100
PE O-18:0_22:6	0.05 ± 0.02	0.04 ± 0.01	0.895	0.07 ± 0.03	0.05 ± 0.03	0.525
PE P-18:0_22:6	0.22 ± 0.13	0.14 ± 0.07	0.364	0.27 ± 0.13	0.10 ± 0.03	0.082

PE 18:1_22:6	0.06 ± 0.01	0.07 ± 0.02	0.682	0.10 ± 0.007	0.05 ± 0.01	0.210
LPE_22:6	0.10 ± 0.04	0.09 ± 0.02	0.895	0.11 ± 0.04	0.06 ± 0.02	0.082
PE-ARA						
PE 16:1_20:4	0.12 ± 0.05	0.08 ± 0.04	0.246	0.28 ± 0.27	0.06 ± 0.03	0.082
PE 16:0_20:4	3.64 ± 1.13	4.04 ± 2.56	>0.999	4.77 ± 1.34	4.88 ± 2.48	0.950
PE O-16:0_20:4	0.44 ± 0.16	0.32 ± 0.22	0.290	0.37 ± 0.25	0.31 ± 0.16	0.936
PE P-16:0_20:4	1.45 ± 0.51	0.89 ± 0.79	0.157	1.54 ± 0.74	0.85 ± 0.34	0.191
PE 18:0_20:4	2.89 ± 0.61	2.12 ± 1.52	0.383	3.92 ± 0.27	2.87 ± 0.76	0.242
PE O-18:0_20:4	0.36 ± 0.18	0.21 ± 0.15	0.290	0.50 ± 0.17	0.27 ± 1.70	0.191
PE P-18:0_20:4	1.30 ± 0.85	0.59 ± 0.55	0.364	1.32 ± 1.00	0.58 ± 0.17	0.386
PE 18:1_20:4	0.40 ± 0.19	0.39 ± 0.20	>0.999	0.48 ± 0.11	0.41 ± 0.20	0.480
PE 18:2_20:4	0.05 ± 0.02	nd		nd	nd	
LPE_20:4	0.85 ± 0.43	0.45 ± 0.33	0.246	1.28 ± 0.56	0.38 ± 0.32	0.100

20:4 = ARA, 22:6 = DHA, PC = phosphatidylcholine, PE = phosphatidylethanolamine
LPC = Lysophosphtidylcholine, LPE = lysophosphatidylethanolamine
In grey all "O" = ether, "p" = plasmalogen species, nd = not detected
Values are the mean ± SD. In bold p value < 0.05 after FDR calculation.

Table S5: Effect of maternal obesity on relative abundance of specific enzymes involved in the phospholipid pathway (*de novo* and remodeling pathways) in placental homogenates regarding the fetal sex.

	**Female**			**Male**		
	**Ctr (n=10)**	**Ob (n=11)**	***P***	**Ctr (n=8)**	**Ob (n=10)**	***P***
GPAT3	1 ± 0.29	1.16 ± 0.28	0.251	1 ± 0.31	1.22 ± 1.21	0.515
AGPAT2	1 ± 0.65	1.24 ± 0.84	0.552	1 ± 0.31	1.01 ± 0.63	0.959
AGPAT4	1 ± 0.30	1.11 ± 0.40	0.468	1 ± 0.21	1.10 ± 0.35	0.762

Values are the mean ± SD, expressed as fraction of the control mean in female and male group. The control (Ctr) and Obese (Ob) groups were compared by the Mann-Whitney U test.

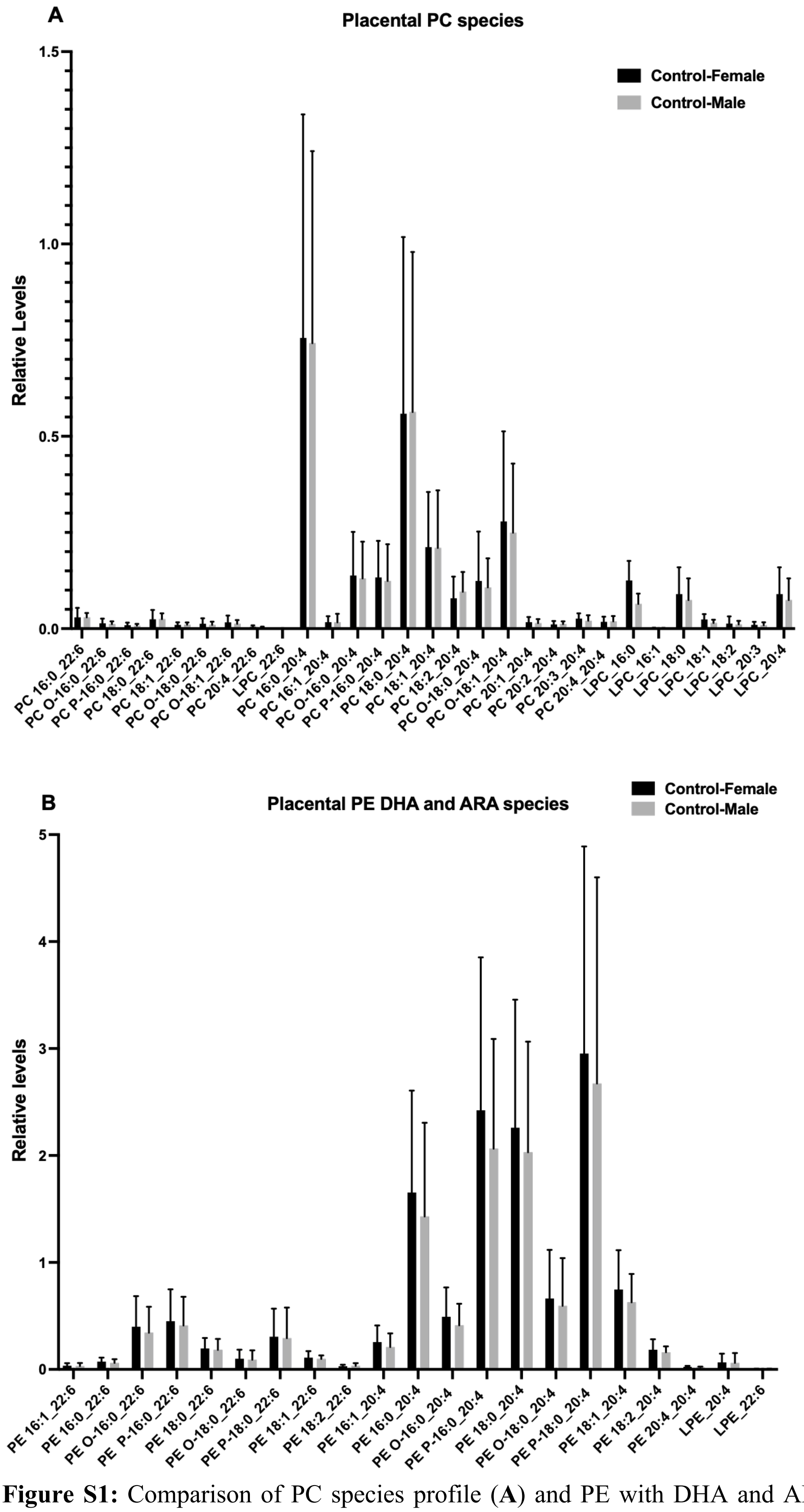

Figure S1: Comparison of PC species profile (**A**) and PE with DHA and ARA profile (**B**) between females and males in control placentas. Control-Female (n=9) and Control-Male (n=7). Variable levels

are expressed in mean ± SD and significant differences pass at false-discovery rate-adjusted p value < 0.05 (Benjamini-Hochberg false discovery rate adjustment to account for multiple testing).

A

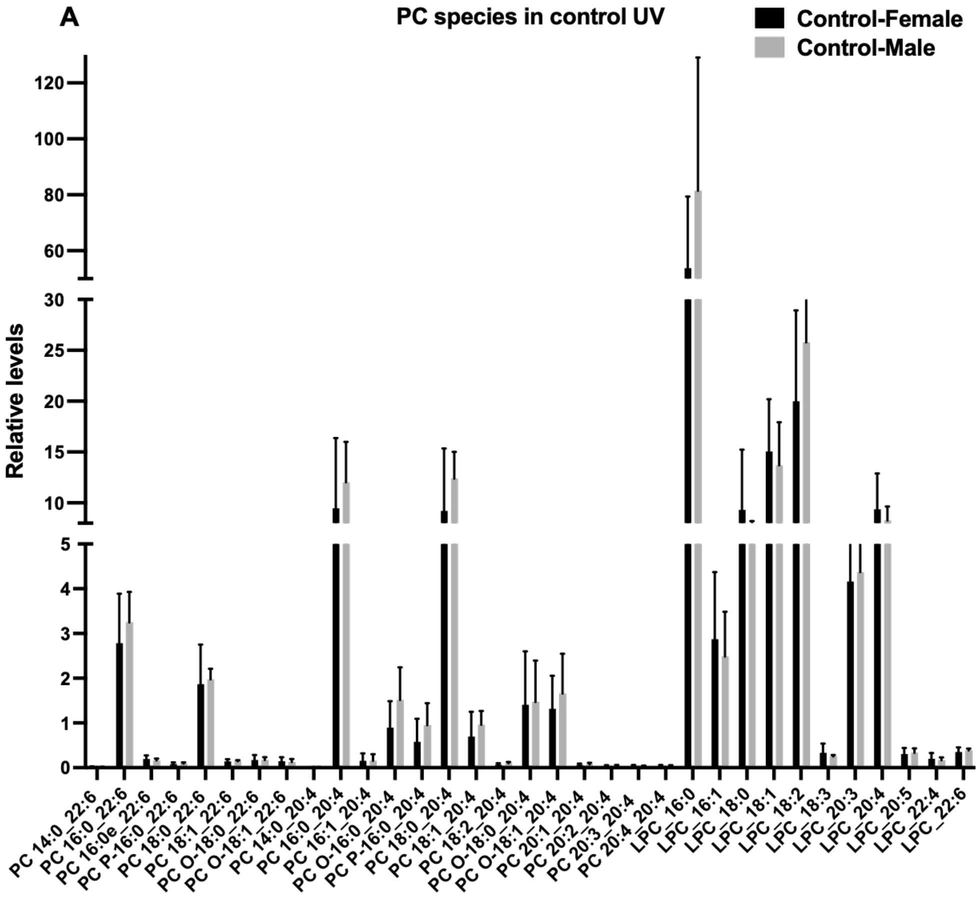

PC species in control UV
Control-Female
Control-Male
Relative levels
PC 14:0_22:6
PC 16:0_22:6
PC 16:0e_22:6
PC P-16:0_22:6
PC 18:0_22:6
PC 18:1_22:6
PC O-18:0_22:6
PC O-18:1_22:6
PC 14:0_20:4
PC 16:0_20:4
PC 16:1_20:4
PC O-16:0_20:4
PC P-16:0_20:4
PC 18:0_20:4
PC 18:1_20:4
PC 18:2_20:4
PC O-18:0_20:4
PC O-18:1_20:4
PC 20:1_20:4
PC 20:2_20:4
PC 20:3_20:4
PC 20:4_20:4
LPC_16:0
LPC_16:1
LPC_18:0
LPC_18:1
LPC_18:2
LPC_18:3
LPC_20:3
LPC_20:4
LPC_20:5
LPC_22:4
LPC_22:6

B

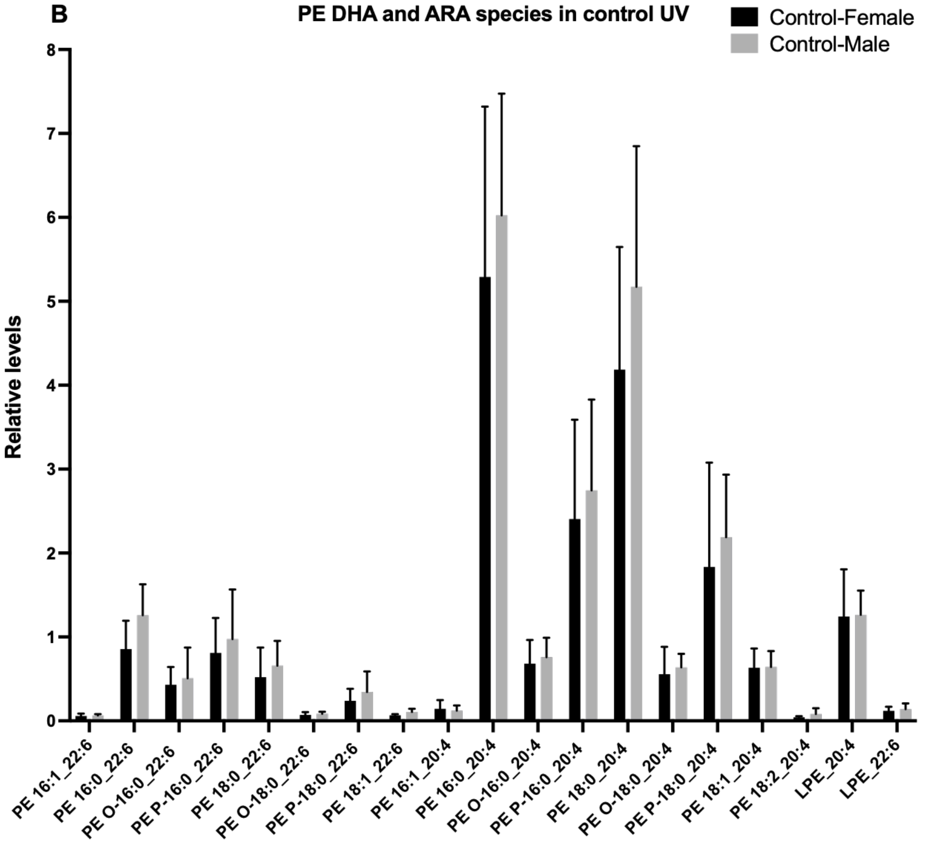

PE DHA and ARA species in control UV
Control-Female
Control-Male
Relative levels
PE 16:1_22:6
PE 16:0_22:6
PE O-16:0_22:6
PE P-16:0_22:6
PE 18:0_22:6
PE O-18:0_22:6
PE P-18:0_22:6
PE 18:1_22:6
PE 16:1_20:4
PE 16:0_20:4
PE O-16:0_20:4
PE P-16:0_20:4
PE 18:0_20:4
PE O-18:0_20:4
PE P-18:0_20:4
PE 18:1_20:4
PE 18:2_20:4
LPE_20:4
LPE_22:6

Figure S2: Comparison of all PC species in particular containing DHA and ARA (**A**) and all PE species (**B**) between females and males in control placentas. Control-Female (n=9) and Control-Male (n=7). Variable levels are the mean ± SD and significant differences pass at false-discovery rate-adjusted p value < 0.05 (Benjamini-Hochberg false discovery rate adjustment to account for multiple testing).
